# Experimental Evidence on Acupuncture Targeting Ferroptosis for Neurological Function Improvement in Cerebral Stroke: A Systematic Review and Meta‐Analysis

**DOI:** 10.1002/brb3.70507

**Published:** 2025-08-21

**Authors:** Wenyu Zhang, Xiaoxi Liu, Xuyang Feng, Donglei Lu, Ruiyu Li, Haizhen Guo, Kun Nie, Xuezhu Zhang

**Affiliations:** ^1^ First Teaching Hospital of Tianjin University of Traditional Chinese Medicine Tianjin China; ^2^ National Clinical Research Center for Chinese Medicine Acupuncture and Moxibustion Tianjin China; ^3^ Tianjin University of Traditional Chinese Medicine Tianjin China; ^4^ Graduate School, Beijing University of Chinese Medicine Beijing China; ^5^ College of Sports Training, Wuhan Sports University Wuhan China; ^6^ Beijing University of Chinese Medicine Shenzhen Hospital (Longgang) Shenzhen China; ^7^ Tianjin University of Traditional Chinese Medicine, Second Affiliated Hospital Tianjin China

**Keywords:** ACSL4, acupuncture, BDNF, cerebral stroke, ferroptosis, GPX4, MDA, meta‐analysis, neurological function, ROS

## Abstract

**Background:**

Acupuncture, a traditional Chinese medicinal practice, has been studied for its ability to regulate ferroptosis and improve neurological function. This systematic review aimed to evaluate the evidence about the effects of acupuncture on ferroptosis and the improvement of neurological function in animal models of cerebral stroke.

**Methods:**

The research was conducted in accordance with the preferred reporting items for systematic reviews and meta‐analyses (PRISMA 2020) guidelines. We conducted a literature search in web of science, Embase, Ebsco, PubMed, Cochrane library, China biomedical literature database (CBM), China national knowledge infrastructure (CNKI), Wanfang database (WF), and VIP database for Chinese technical periodicals, covering pertinent studies up to May 30, 2024. The inclusion criteria covered experimental animal models of cerebral stroke that underwent acupuncture interventions (electroacupuncture [EA], manual acupuncture [MA], or moxibustion [Moxi]). The primary outcomes consisted of neurological function scores (Longa, modified neurological severity score [mNSS], Zausinger, Garcia, Ludmila Belayev scores, and levels of brain‐derived neurotrophic factor [BDNF]). Secondary outcomes encompassed iron metabolism (iron ion content, ferritin heavy chain 1 [FTH1], transferrin receptor 1 [TFR1]), lipid peroxidation (malondialdehyde [MDA], reactive oxygen species [ROS]), antioxidative parameters (glutathione peroxidase 4 [GPX4], glutathione [GSH], superoxide dismutase [SOD]), and ferroptosis markers (long‐chain acyl‐CoA synthetase 4 [ACSL4]). Two authors independently evaluated the methodological quality of the included studies utilizing the collaborative approach to meta‐analysis and review of animal data from experimental studies (CAMARADES) 10‐item checklist.

**Results:**

The meta‐analysis indicated that acupuncture markedly enhanced neurological function scores (Longa, Longa [mNSS], Zausinger, Garcia, Ludmila Belayev) and elevated levels of BDNF, GSH, GPX4, and SOD in animal models of cerebral stroke. Furthermore, it significantly reduced the levels of cerebral iron, FTH1, TFR1, MDA, ROS, and ACSL4.

**Conclusions:**

Acupuncture effectively inhibits ferroptosis and enhances neurological function in animal models of cerebral stroke by modulating brain iron metabolism, decreasing lipid peroxidation, and improving brain antioxidant capacity.

## Graphical abstract

A systematic experimental analysis demonstrates that acupuncture promotes brain repair by multi‐target, multi‐pathway inhibition of ferroptosis, significantly enhancing neurological recovery in experimental stroke models. It effectively modulates iron overload, lipid peroxidation, and antioxidant defenses, leading to comprehensive neurological improvements. This study elucidates acupuncture's ferroptosis‐inhibiting mechanism, providing a critical neuroprotective strategy for stroke intervention.

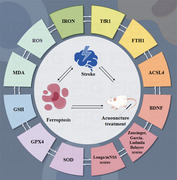

## 1 Introduction

Cerebral stroke (also known as “stroke”) is an acute cerebrovascular disease characterized by the sudden rupture or obstruction of cerebral blood arteries, resulting in decreased blood supply to the brain and consequent brain tissue destruction (GBD 2019 Diseases and Injuries Collaborators 2020). It includes both hemorrhagic and ischemic types, with common symptoms such as headache, vomiting, altered consciousness, coma, hemiplegia, and dysphagia (GBD 2019 Stroke Collaborators 2021). Globally, stroke is the second leading cause of death and disability. Despite advancements in medical care, stroke remains one of the leading causes of mortality worldwide, posing a significant threat to human health. Statistics reveal that between 1990 and 2019, the worldwide incidence of stroke‐related fatalities rose by 43%, culminating in 6.55 million deaths, while almost 101 million individuals suffered a stroke in 2019. In a large, nationally representative sample of adults aged 40 years or older, the estimated prevalence, incidence, and mortality rate of stroke in China in 2020 were 2.6%, 505.2 per 100,000 person‐years, and 343.4 per 100,000 person‐years, respectively (Tu et al. 2023). Extensive neuronal loss in the early stages of stroke is a pivotal determinant of mortality and disability, while the financial burden of treatment imposes a considerable strain on stroke patients and their families (Wang et al. 2020).

**FIGURE 1:**
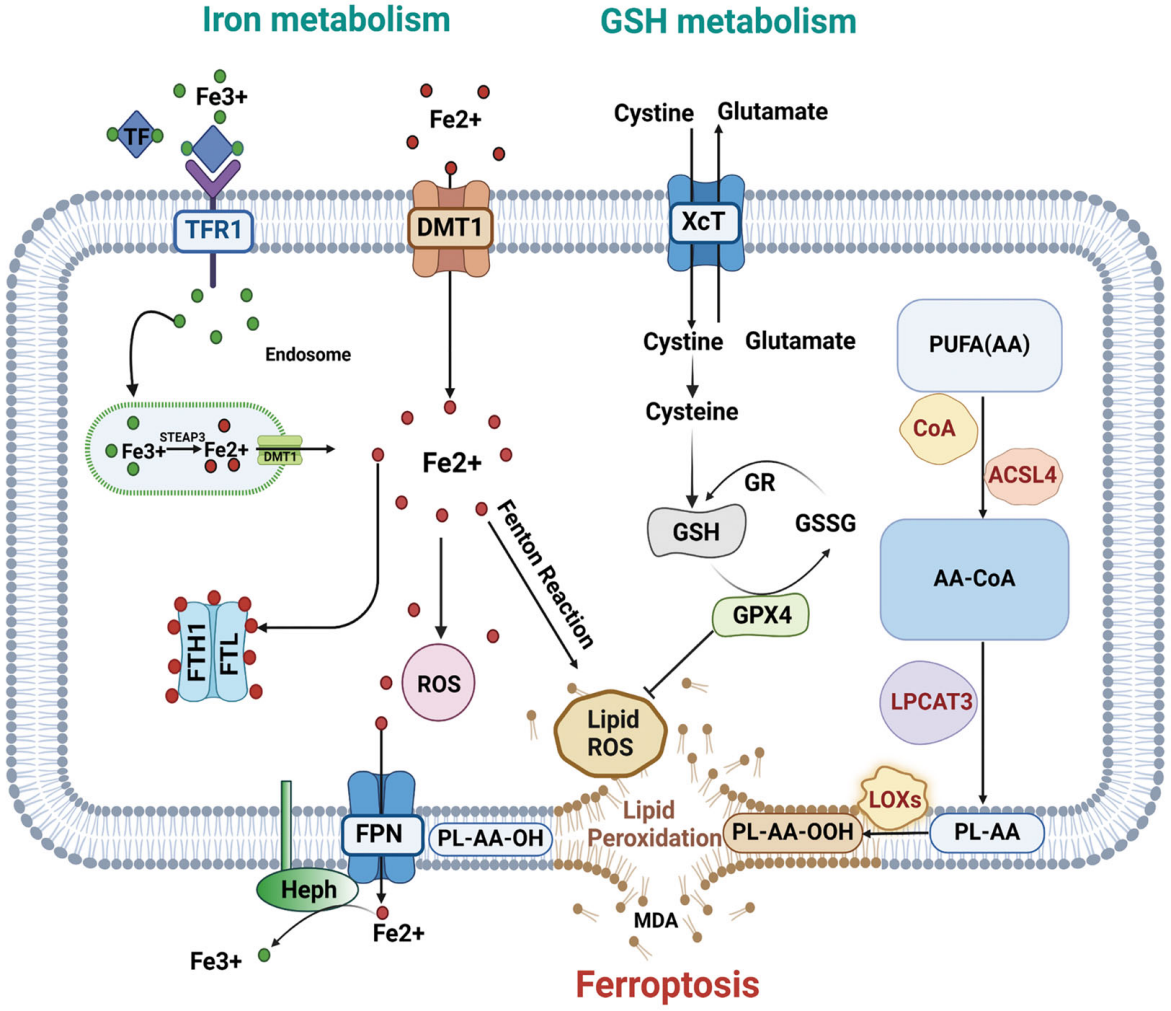
Mechanism of ferroptosis. Note: Iron (Fe^3+^) binds to transferrin (TF) and is imported into cells via the membrane protein TFR1. It is then stored in endosomes, where it undergoes reduction to Fe^2^⁺ by the six‐transmembrane epithelial antigen of the prostate 3 (STEAP3). Fe^2+^ is transported out of endosomes by divalent metal transporter 1 (DMT1). Most Fe^2+^ is stored in cytosolic ferritin (composed of FTH1) and ferritin light chain (FTL), and its export is mediated by the membrane protein ferroportin 1 (FPN1). Excess Fe^2+^ can generate ROS via the Fenton reaction or iron‐catalyzed enzymatic pathways, and participate in the synthesis of lipoxygenases (LOXs) to catalyze the oxidation of polyunsaturated fatty acids (PUFAs), thereby inducing ferroptosis. The X_c_^−^ system imports cystine and exports glutamate. Cystine is reduced to cysteine, which is then used to produce GSH. GSH is a substrate for the synthesis of GPX4, both of which can repair cell membrane lipids. GPX4 reduces lipid hydroperoxides (L‐OOH) to lipid alcohols (L‐OH). In summary, ferroptosis results from iron overload, disruptions in the antioxidant defense system, and the excessive accumulation of lipid peroxides.

Ferroptosis is an iron‐dependent, lipid peroxidation‐mediated form of cell death characterized by metabolic pathway disruptions within cells. Excessive iron ion and reactive oxygen species (ROS) accumulation causes membrane phospholipid (mPL) peroxidation and cell membrane damage. Antioxidant systems, such as reduced glutathione (GSH) and glutathione peroxidase 4 (GPX4), become less effective in eliminating excess lipid peroxides (Jiang et al. 2021; Liang et al. 2022; Li et al. 2020). Consequently, the cell dies due to membrane rupture. The pathological mechanisms of ferroptosis can be summarized into three main points (Liu et al. 2022):(1) iron overload and dysregulated iron metabolism, (2) lipid peroxidation damage, and (3) imbalance in the antioxidant system (Figure 1).According to research, ferroptosis plays a critical role in the pathogenesis of stroke (Tian et al. 2024). Under stroke pathogenic conditions, rupture of the blood‐brain barrier (BBB) causes a rapid influx of iron ions into the brain parenchyma (DeGregorio‐Rocasolano et al. 2019). During ischemia, neurons are exposed to high levels of extracellular iron, which play an important part in stroke‐induced neuropathology (Weiland et al. 2019). Neuronal ferroptosis occurs after a stroke. Preventing ferroptosis may protect neurons from death, reduce secondary brain injury, and improve patient outcomes, indicating that ferroptosis is a major potential target for stroke intervention (Zille et al. 2017; Li et al. 2018; Li et al. 2017; Tuo et al. 2017; Alim et al. 2019).

Acupuncture, an important component of traditional Chinese medicine, dates back thousands of years and is known for its holistic approach and balance‐regulating therapies. Acupuncture aims to regulate the flow of “Qi” throughout the body and facilitate gradual recovery by stimulating specific acupoints (Lundeberg and Kurosawa 2010; Wang et al. 2023; Wen et al. 2021). Acupuncture has recently attained worldwide acknowledgment, especially in the treatment of neurological illnesses, such as stroke (Chavez et al. 2017). Acupuncture improves neurological function following a stroke and suppresses ferroptosis via multiple pathways, thereby protecting neurons and reducing brain damage (Lang et al. 2024; Wang et al. 2023; Zhu et al. 2024). In rat models of intracerebral hemorrhage (ICH), acupuncture reduces neuronal death, inflammation, and ferroptosis by downregulating miR‐23a‐3p levels, increasing GSH/GPX4 expression, lowering iron and malondialdehyde (MDA) levels, and decreasing ROS levels (Kong et al. 2021). Acupuncture can also activate the p62/Keap1/Nrf2 pathway, which in turn promotes the recovery of neurological function following hemorrhagic stroke by increasing the expression of GPX4 and ferritin heavy chain 1 (FTH1), decreasing iron levels, and reducing lipid peroxidation damage in ICH rat models (Li et al. 2022). In a rat model of chronic unpredictable mild stress (CUMS), acupuncture can reverse MDA, superoxide dismutase (SOD), GSH, and GSH‐Px levels in rat serum and partially restore Sirt1, Nrf2, HO‐1, and GPX4 levels in the rat hippocampus, thereby reducing ferroptosis by inhibiting the increase in hippocampal iron content (Shen et al. 2024). Furthermore, acupuncture could regulate the ACSL4‐15LO1 pathway, significantly decreasing MDA and GSSG levels as well as ACSL4‐15‐LO1 protein expression in MCAO/R rat models, reducing lipid peroxidation in ferroptosis, and alleviating ischemia‐reperfusion injury (Tang et al. 2023). Nonetheless, differences in the selection of stroke animal models, methodologies used, and the criteria for neuroprotective outcome indicators have resulted in variations in the quality of previous experimental studies (Locker 2004). These differences may significantly contribute to the failure to translate results from experimental animal studies to clinical research, resulting in negative outcomes (Ioannidis et al. 2014; Levy 2012). Consequently, it is essential to systematically verify the efficacy of acupuncture and clarify its potential mechanisms in animal models. This comprehensive approach will help bridge the gap between experimental and clinical research and enhance our understanding of acupuncture's therapeutic potential in alleviating neurological dysfunction after stroke.

## 2 Results

### 2.1 Systematic Search, Selection, and Data Extraction

**FIGURE 2:**
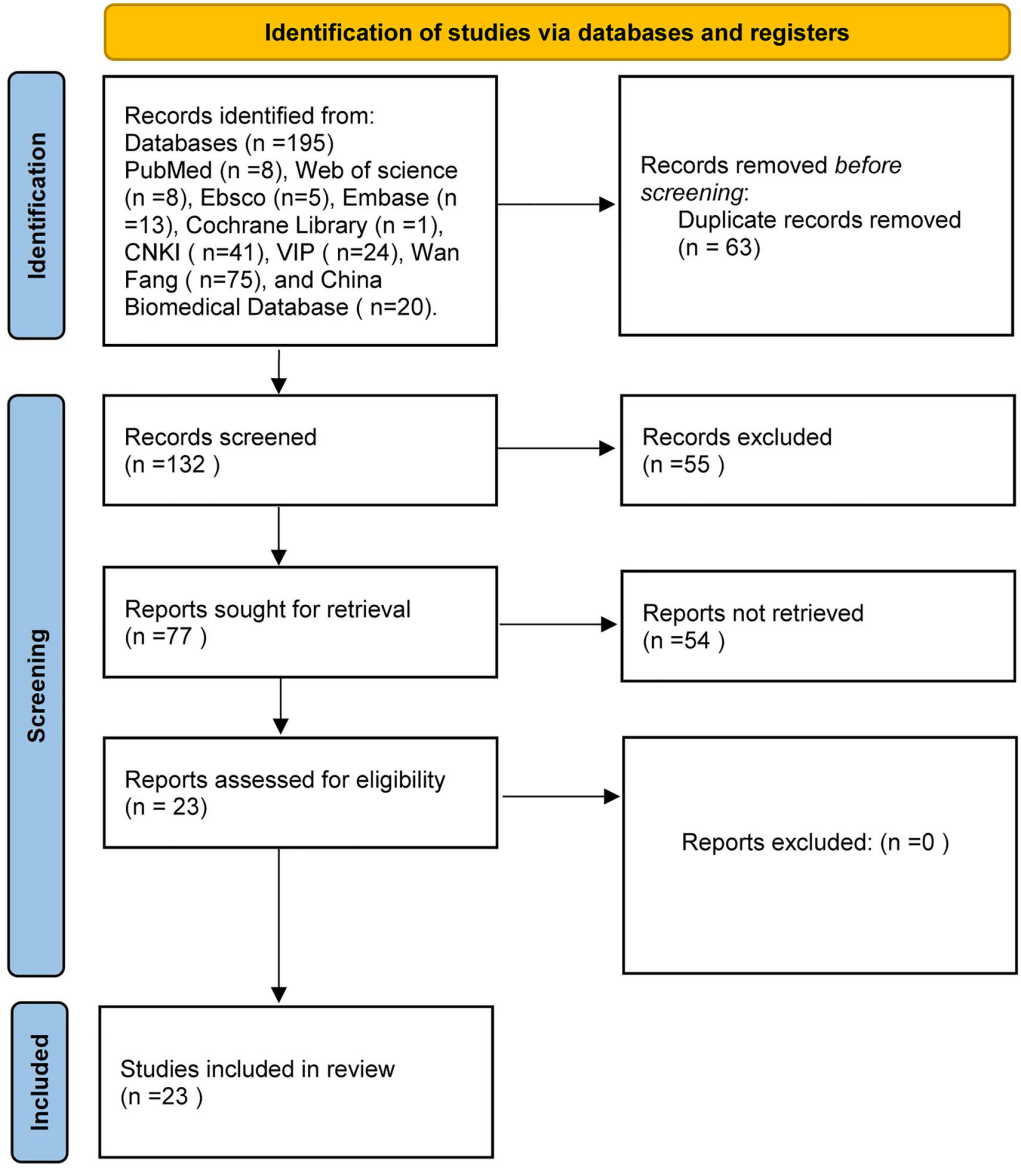
Flow chart of systematic search, selection, and data extraction according to PRISMA.

The initial database search identified 195 potential articles: 8 from PubMed, 8 from Web of Science, 13 from Embase, 5 from Ebsco, 1 from Cochrane Library, 41 from China national knowledge infrastructure (CNKI), 75 from Wanfang, 24 from VIP, and 20 from the China Biomedical Literature Database. After deleting duplicates, 132 unique articles remained. After reviewing the titles and abstracts, 55 publications were removed. After full‐text assessment, 54 more articles were excluded. Ultimately, 23 articles met the inclusion criteria for this systematic review and meta‐analysis. Figure 2 illustrates the complete procedure of literature screening.

### 2.2 Characteristics of Included Studies

A total of 23 studies with 774 experimental animals were included. Five different methods were used to assess neurological function in these experimental animals: Longa score (5 studies, 157 animals) (Chen et al. 2012; Lin et al. 2015; Tao et al. 2016; Wu et al. 2023; Sun et al. 2022), Longa, modified neurological severity score (mNSS) score (3 studies, 468 animals) (Chen et al. 2022; Yang 2024; Liang 2023), Zausinger score (2 studies, 60 animals) (Wang et al. 2024; Gao and Yang 2023), Ludmila Belayev score (3 studies, 298 animals) (Li et al. 2022; Dai et al. 2024; Li et al. 2021), and Garcia score (3 studies, 136 animals) (Kim et al. 2012; Zhang et al. 2023; Wang et al. 2022). These methods evaluated neurological improvement in stroke animal models subjected to acupuncture from different perspectives. Five trials (165 animals) (Chen et al. 2012; Tao et al. 2016; Sun et al. 2022; Kim et al. 2012; Zheng et al. 2020) measured levels of brain‐derived neurotrophic factor (BDNF) to examine acupuncture's effect on neuroregeneration and repair. For ferroptosis, nine studies (680 animals) (Wu et al. 2023; Chen et al. 2022; Yang 2024; Liang 2023; Gao and Yang 2023; Li et al. 2022; Zhang et al. 2023; Wang et al. 2022; Zhang et al. 2024) reported brain tissue iron content, three studies (78 animals) (Chen et al. 2022; Wang et al. 2024; Wang et al. 2023) reported transferrin receptor 1 (TfR1) levels, and two studies (132 animals) (Li et al. 2022; Zhang et al. 2024) reported FTH1. These studies investigated how acupuncture affected iron metabolism and ferroptosis after stroke. Twelve studies (834 animals) (Lin et al. 2015; Wu et al. 2023; Yang 2024; Wang et al. 2024; Gao and Yang 2023; Li et al. 2022; Dai et al. 2024; Li et al. 2021; Zhang et al. 2023; Wang et al. 2022; Zhang et al. 2024; Lin et al. 2024) reported MDA content in brain tissue, and four studies (328 animals) (Yang 2024; Liang 2023; Wang et al. 2022; Lin et al. 2024) reported ROS levels. These measures explored acupuncture's effect on lipid peroxidation and its relevance to ferroptosis in stroke. Seven studies (428 animals) (Lin et al. 2015; Wu et al. 2023; Liang 2023; Wang et al. 2024; Gao and Yang 2023; Dai et al. 2024; Zhang et al. 2023) reported GSH levels; six studies (228 animals) (Lin et al. 2015; Wu et al. 2023; Wang et al. 2024; Gao and Yang 2023; Dai et al. 2024; Zhang et al. 2023) reported GPX4 levels; and five studies (142 animals) (Lin et al. 2015; Sun et al. 2022; Lin et al. 2024; Liu et al. 2006; Zhang et al. 2014) reported SOD levels. These indicators assessed antioxidation regulation associated with ferroptosis in brain tissue following a stroke using acupuncture. Three studies (270 animals) (Yang 2024; Zhang et al. 2023; Wang et al. 2023) reported ferroptosis marker ACSL4 levels.

For animal models, 2 studies (8.7%) (Zheng et al. 2020; Wang et al. 2023) used the middle cerebral artery occlusion (MCAO) model (Longa et al. 1989), 12 studies (52.2%) (Chen et al. 2012; Lin et al. 2015; Tao et al. 2016; Wu et al. 2023; Yang 2024; Liang 2023; Wang et al. 2024; Kim et al. 2012; Zhang et al. 2023; Wang et al. 2022; Zhang et al. 2024; Lin et al. 2024) used the middle cerebral artery occlusion/reperfusion (MCAO/R) model (Longa et al. 1989), 5 studies (21.7%) (Chen et al. 2022; Gao and Yang 2023; Li et al. 2022; Dai et al. 2024; Li et al. 2021) used the intracerebral hemorrhage (ICH) model (MacLellan et al., 2008 Mar), 2 studies (8.7%) (Liu et al. 2006; Zhang et al. 2014) used the multi‐infarct dementia (MID) model (Kaneko et al. 1985; Chen et al. 1994), 1 study (4.3%) (Zuo et al. 2017) used the four‐vessel occlusion (4‐VO) model (Pulsinelli and Brierley 1979), and 1 study (4.3%) (Sun et al. 2022) used the post‐stroke depression (PSD) model (Willner et al. 1987; Willner 1990).

Interventions included EA in 13 studies (56.5%) (Chen et al. 2012; Lin et al. 2015; Tao et al. 2016; Wu et al. 2023; Chen et al. 2022; Liang 2023; Wang et al. 2024; Gao and Yang 2023; Kim et al. 2012; Zheng et al. 2020; Wang et al. 2023; Lin et al. 2024; Zuo et al. 2017), MA in 9 studies (39.1%) (Sun et al. 2022; Yang 2024; Li et al. 2022; Dai et al. 2024; Li et al. 2021; Wang et al. 2022; Zhang et al. 2024; Liu et al. 2006; Zhang et al. 2014), and Moxi in 1 study (4.3%) (Zhang et al. 2023). Finally, 8 studies (34.8%) (Chen et al. 2012; Lin et al. 2015; Tao et al. 2016; Wu et al. 2023; Liang 2023; Wang et al. 2024; Wang et al. 2022; Wang et al. 2023) reported infarct volume. The study characteristics are summarized in Table S1.

### 2.3 Study Quality

The quality scores of the included studies ranged from 6 to 9 points (out of a total of 10). Among the 23 studies, 1 study (4.3%) received a score of 6, 11 studies (47.8%) received a score of 7, 7 studies (30.4%) received a score of 8, and 4 studies (17.4%) received a score of 9 (Table 1). All 23 studies (100%) were published in peer‐reviewed journals. Twenty‐one studies (91.3%) described temperature control. All 23 studies (100%) reported random allocation to treatment groups. Five studies (21.7%) described blinded induction of the stroke model. All 23 studies (100%) used anesthetics without significant intrinsic neuroprotective activity. All 23 studies (100%) employed appropriate animal models. All studies (100%) mentioned compliance with animal welfare regulations. All studies (100%) included statements of no potential conflicts of interest. All studies (100%) included funding statements. No studies reported blinded outcome assessment or sample size calculations.

**TABLE 1:** Risk of bias of the included studies.

Study	A	B	C	D	E	F	G	H	I	J	Total
Wang 2023	√	√	√			√	√	√	√	√	8
Chen 2022	√	√	√			√	√	√	√	√	8
Wu 2023	√	√	√			√	√	√		√	7
Li 2022	√	√	√	√		√	√	√	√	√	9
Zhang 2024	√		√			√	√	√	√	√	7
Tao 2015	√	√	√	√		√	√	√	√	√	9
Kim 2012	√	√	√	√		√	√	√	√	√	9
Wang 2024	√	√	√			√	√	√		√	7
Dai 2024	√	√	√			√	√	√		√	7
Yang 2023	√		√			√	√	√		√	6
Gao 2023	√	√	√			√	√	√		√	7
Wang 2022	√	√	√			√	√	√		√	7
Liang 2022	√	√	√			√	√	√		√	7
Li 2021	√	√	√			√	√	√		√	7
Zhang 2023	√	√	√			√	√	√	√	√	8
Zhang 2014	√	√	√			√	√	√	√	√	8
Zuo 2017	√	√	√			√	√	√		√	7
Lin 2024	√	√	√	√		√	√	√	√	√	9
Lin 2015	√	√	√			√	√	√	√	√	8
Liu 2005	√	√	√			√	√	√		√	7
Sun 2022	√	√	√			√	√	√		√	7
Chen 2012	√	√	√	√		√	√	√		√	8
Zheng 2020	√	√	√			√	√	√	√	√	8

*Note*: Studies fulfilling the criteria of: A: peer reviewed publication; B: control of temperature; C: random allocation to treatment or control; D: blinded induction of model; E: blinded assessment of outcome; F: use of anesthetic without significant intrinsic neuroprotective activity; G: appropriate animal model (cerebral hemorrhage or cerebral ischemia); H: compliance with animal welfare regulations; I: statement of potential conflict of interests. J: funding Statement.

### 2.4 Effectiveness

#### 2.4.1 Overall Effect of Acupuncture on Neurological Function Improvement

**FIGURE 3:**
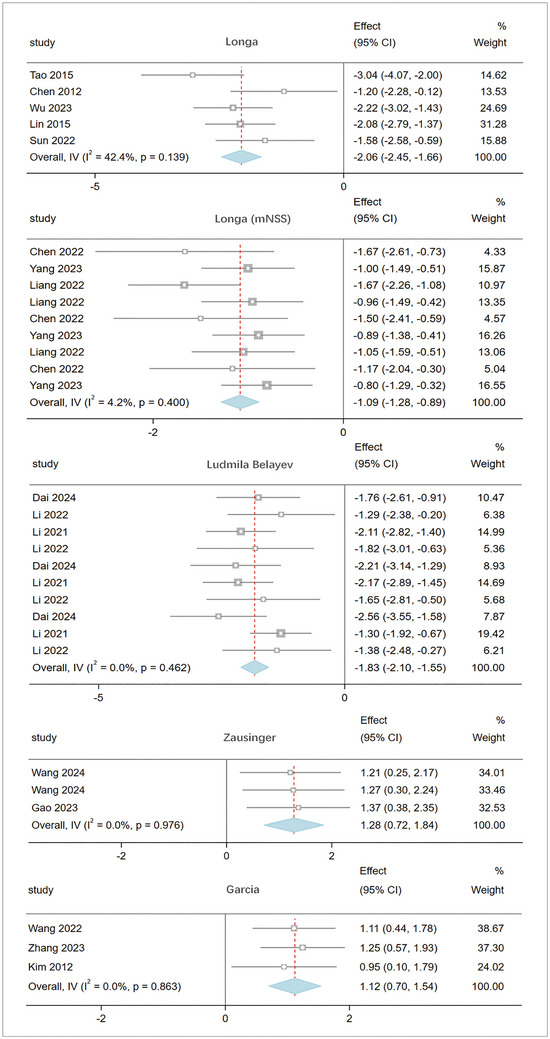
Pooled result of neurological function score based on acupuncture therapy in experimental cerebral stroke. Note: The neurological function in the acupuncture group has significantly improved, and the results are consistent across different evaluation methods.

The meta‐analysis of 17 studies involving 1,119 rats evaluated neurological function scores. The pooled results (Figure 3) indicate that, based on the Longa score (Longa et al. 1989), the Longa (mNSS) score (Lu et al. 2003), the Ludmila Belayev score (Belayev et al. 1996), the Zausinger score (Zausinger et al. 2000), and Garcia score (Garcia et al. 1995). The acupuncture group showed significant improvement in neurological deficits after stroke compared to the model group. Heterogeneity was low (I^2^ < 50%), justifying the use of a fixed‐effects model. Longa: I^2^ = 42.4%; MD: ‐2.06, 95% CI [‐2.45, ‐1.66], p = 0.139. Longa (mNSS): I^2^ = 4.2%; MD: ‐1.09, 95% CI [‐1.28, ‐0.89], p = 0.400. Ludmila Belayev: I^2^ = 0%; MD: ‐1.83, 95% CI [‐2.10, ‐1.55], p = 0.462. Zausinger: I^2^ = 0%; MD: 1.28, 95% CI [0.72, 1.84], p = 0.976. Garcia: I^2^ = 0%; MD: 1.12, 95% CI [0.70, 1.54], p = 0.863. These results demonstrate a significant improvement in neurological function in the acupuncture group, with consistent findings across different evaluation methods.

**FIGURE 4:**
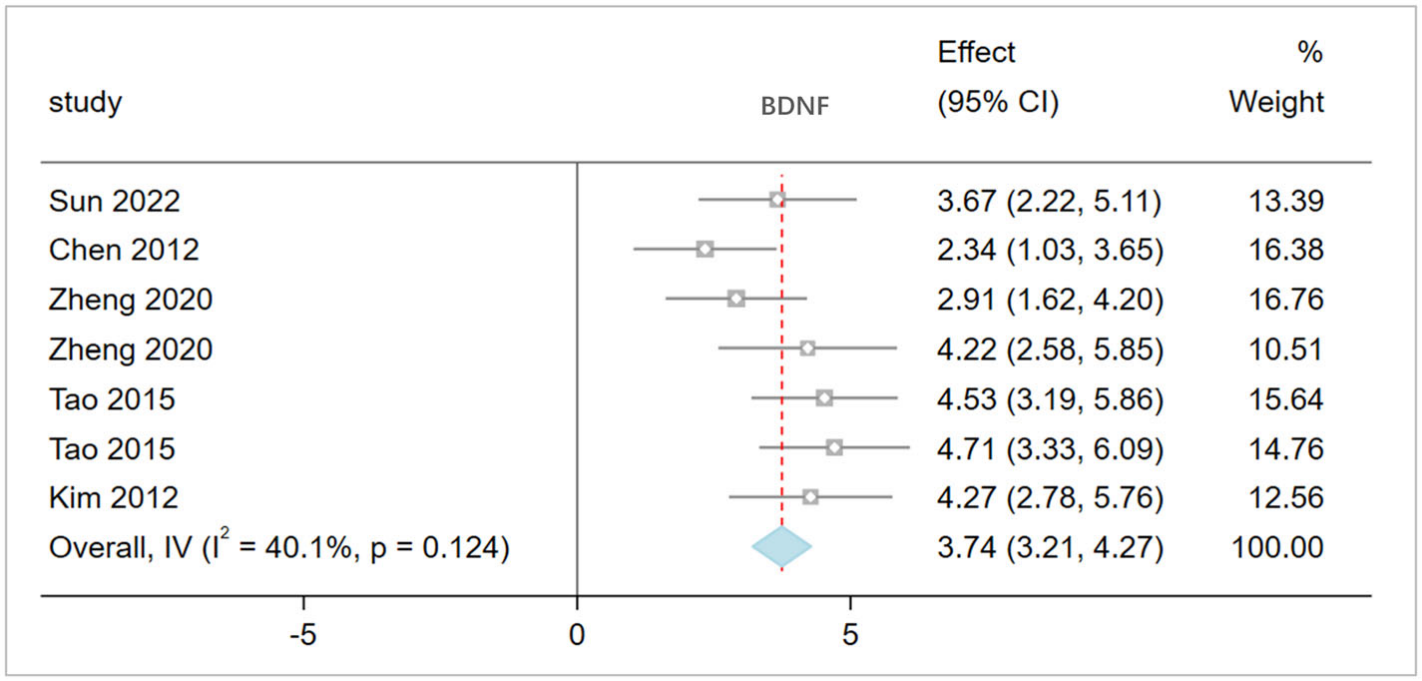
The result of BDNF based on acupuncture therapy in experimental cerebral stroke. Note: The BDNF levels in the acupuncture group were significantly increased.

Neuroregeneration and brain tissue repair: The meta‐analysis of five studies involving 165 rats evaluated brain tissue BDNF levels. The pooled results (Figure 4) show that acupuncture significantly promotes neuroregeneration and tissue repair after stroke, as indicated by BDNF: I^2^ = 40.1%; SMD: 3.74, 95% CI [3.21, 4.27], p = 0.124.

#### 2.4.2 Overall Effect of Acupuncture on Ferroptosis

**FIGURE 5:**
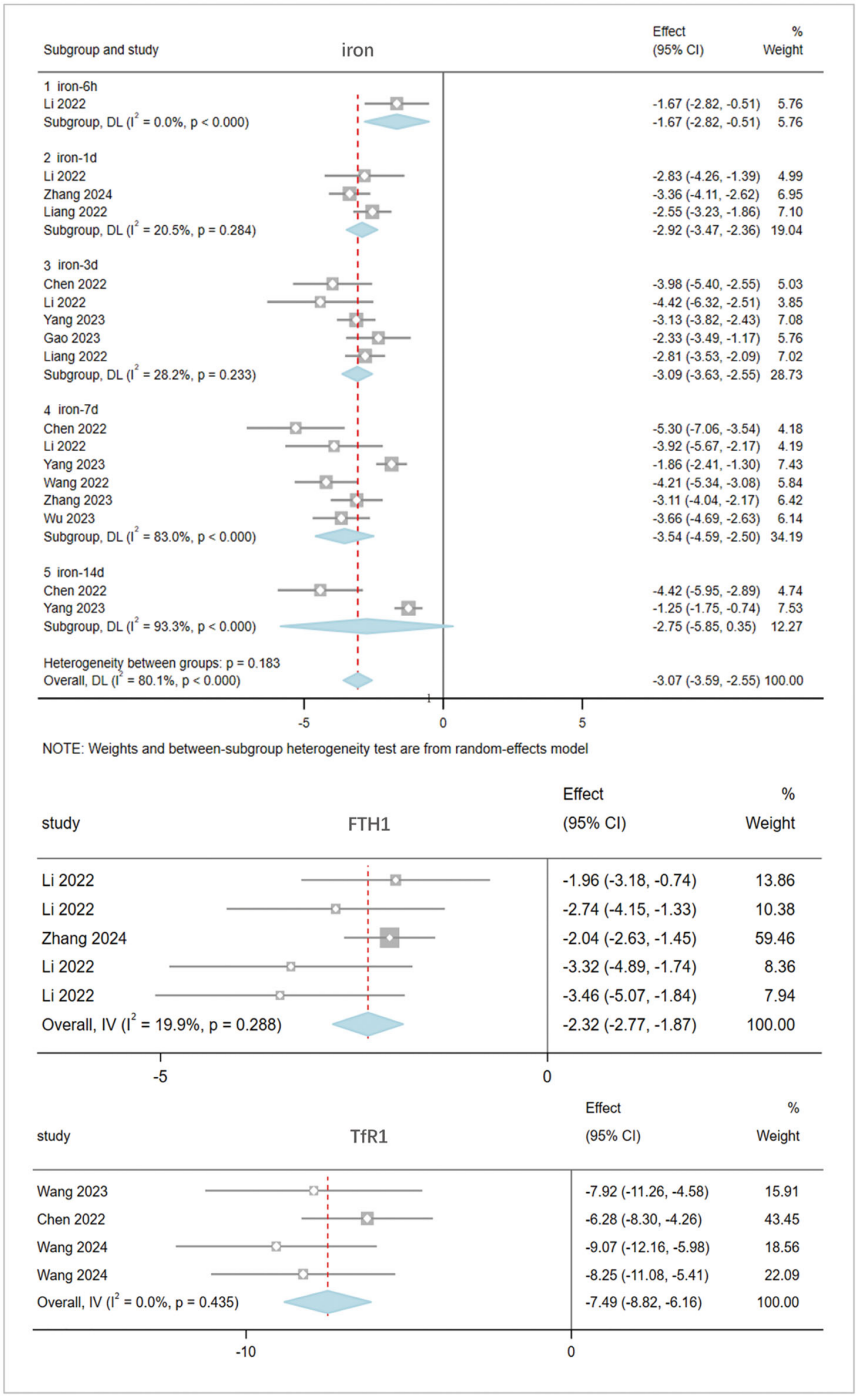
The result of iron, FTH1 and TfR1 based on acupuncture therapy in experimental cerebral stroke. Note: The iron metabolism in the acupuncture group has significantly improved, and the results are consistent across different evaluation methods.

Iron overload and iron metabolism: The meta‐analysis of 26 studies involving 890 rats evaluated brain tissue iron overload and metabolism after stroke. The pooled results (Figure 5) show that, compared to the model group, acupuncture significantly reduces iron ion deposition (lowering iron content) and promotes iron metabolism (reducing FTH1 and TfR1 levels) in brain tissue after stroke, thereby inhibiting ferroptosis. Iron: I^2^ = 80.1%, random‐effects model; SMD: ‐3.07, 95% CI [‐3.59, ‐2.55], p = 0.183. Furthermore, from 6 h to 14 days after stroke, acupuncture significantly reduced brain tissue iron content at various time points (Iron‐6h: I^2^ = 0%; SMD: ‐1.67, 95% CI [‐2.82, ‐0.51], ***p < 0.0001. Iron‐1d: I^2^ = 20.5%, SMD: ‐2.92, 95% CI [‐3.47, ‐2.36], p = 0.284. Iron‐3d: I^2^ = 28.2%, SMD: ‐3.09, 95% CI [‐3.63, ‐2.55], p = 0.233. Iron‐7d: I^2^ = 83.0%, SMD: ‐3.54, 95% CI [‐4.59, ‐2.50], ***p < 0.0001. Iron‐14d: I^2^ = 93.3%, SMD: ‐2.75, 95% CI [‐5.85, 0.35], p < 0.0001). FTH1: I^2^ = 19.9%; SMD: ‐2.32, 95% CI [‐2.77, ‐1.87], p = 0.288. TfR1: I^2^ = 0%; SMD: ‐7.49, 95% CI [‐8.82, ‐6.16], p = 0.435.

**FIGURE 6:**
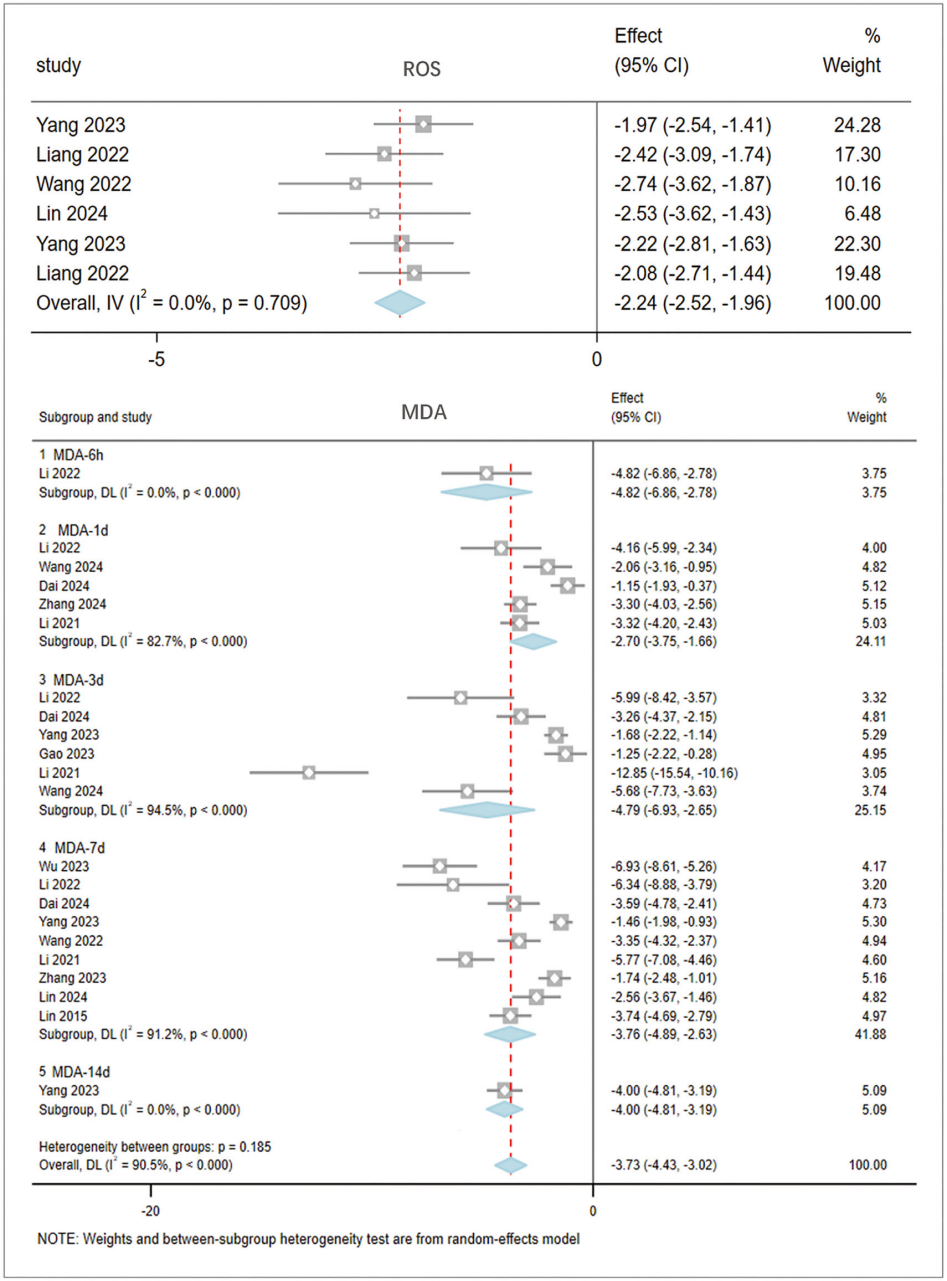
The result of ROS and MDA based on acupuncture therapy in experimental cerebral stroke. Note: The lipid peroxidation in the acupuncture group has significantly reduced, and the results are consistent across different evaluation methods.

Lipid peroxidation: The meta‐analysis of 28 studies involving 1,162 rats evaluated lipid peroxidation in brain tissue after stroke. The pooled results (Figure 6) indicate that, compared to the model group, acupuncture significantly reduces lipid peroxidation in brain tissue (lowering ROS and MDA levels), thereby inhibiting ferroptosis. ROS: I^2^ = 0%; SMD: ‐2.24, 95% CI [‐2.52, ‐1.96], p = 0.709. ROS levels in the brain tissue of the acupuncture group were significantly reduced. MDA: I^2^ = 90.5%, high heterogeneity (I^2^ > 50%), random‐effects model, SMD: ‐3.73, 95% CI [‐4.43, ‐3.02], p = 0.185. In addition, from 6 h to 14 days after stroke, acupuncture significantly reduced brain tissue MDA levels at various time points: MDA‐6h: I^2^ = 0%, SMD: ‐4.82, 95% CI [‐6.86, ‐2.78], ***p < 0.0001. MDA‐1d: I^2^ = 82.7%, SMD: ‐2.70, 95% CI [‐3.75, ‐1.66], ***p < 0.0001. MDA‐3d: I^2^ = 94.5%, SMD: ‐4.79, 95% CI [‐6.93, ‐2.65], ***p < 0.0001. MDA‐7d: I^2^ = 91.2%, SMD: ‐3.76, 95% CI [‐4.89, ‐2.63], ***p < 0.0001. MDA‐14d: I^2^ = 0%, SMD: ‐4.00, 95% CI [‐4.81, ‐3.19], ***p < 0.0001.

**FIGURE 7:**
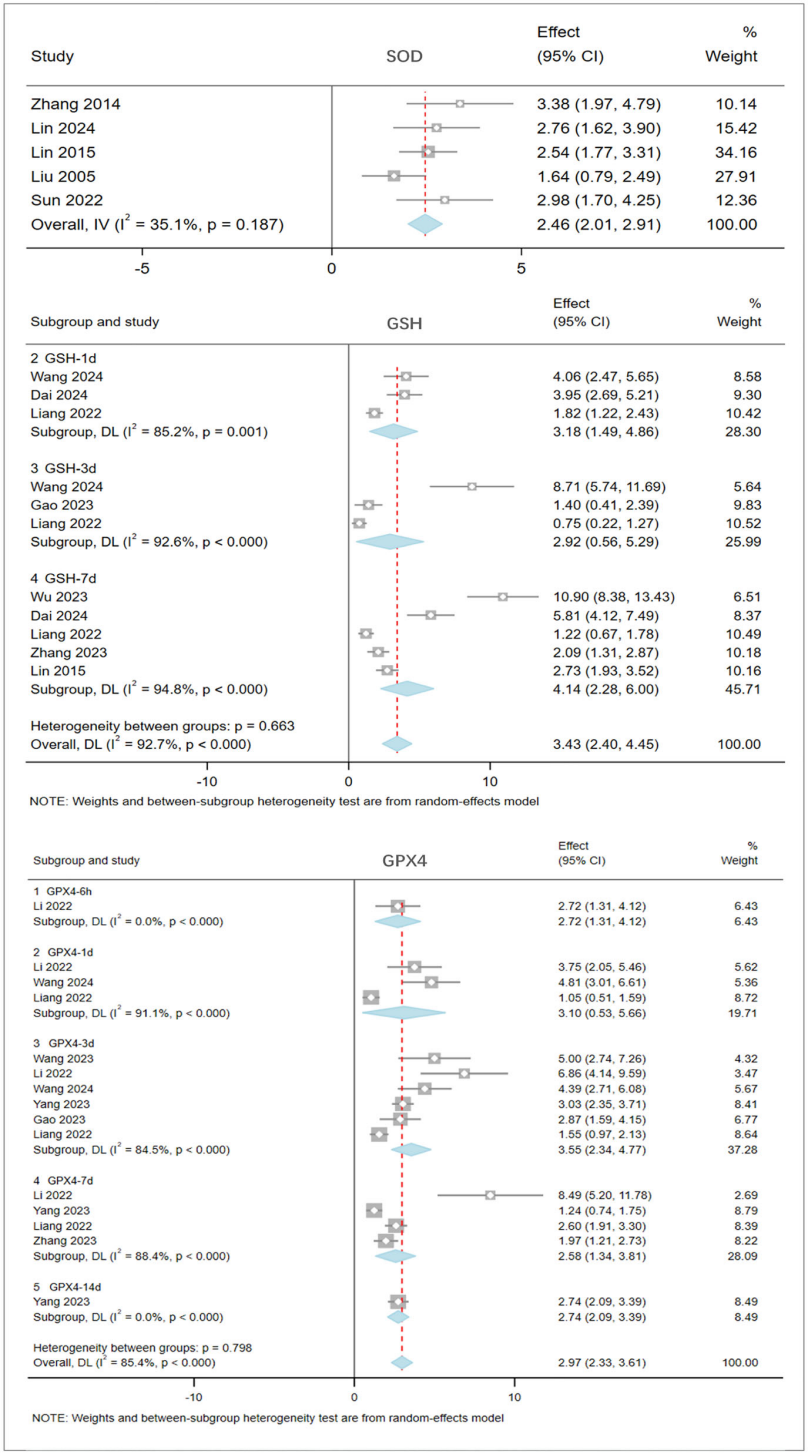
The result of SOD, GSH, and GPX4 based on acupuncture therapy in experimental cerebral stroke. Note: The antioxidant function in the acupuncture group was significantly improved, and the results were consistent across different evaluation methods.

The meta‐analysis of 27 studies involving 798 rats evaluated the antioxidant capacity of brain tissue after stroke. The pooled results (Figure 7) indicate that, compared to the model group, acupuncture significantly enhances the antioxidant capacity (increasing SOD, GSH, and GPX4 levels), thereby inhibiting ferroptosis. SOD: I^2^ = 35.1%, SMD: 2.46, 95% CI [2.01, 2.91], p = 0.187. SOD levels in the brain tissue of the acupuncture group were significantly increased. GSH: I^2^ = 92.7%, high heterogeneity (I^2^ > 50%), random‐effects model, SMD: 3.43, 95% CI [2.40, 4.45], p = 0.663. From 1 to 7 days after stroke, acupuncture significantly increased brain tissue GSH levels at various time points: GSH‐1d: I^2^ = 85.2%, SMD: 3.18, 95% CI [1.49, 4.86], p = 0.001. GSH‐3d: I^2^ = 92.6%, SMD: 2.92, 95% CI [0.56, 5.29], ***p < 0.0001. GSH‐7d: I^2^ = 94.8%, SMD: 4.14, 95% CI [2.28, 6.00], ***p < 0.0001. GPX4: I^2^ = 85.4%, high heterogeneity (I^2^ > 50%), random‐effects model, SMD: 2.97, 95% CI [2.33, 3.61], p = 0.798. From 6 h to 14 days after stroke, acupuncture significantly increased brain tissue GPX4 levels at various time points: GPX4‐6h: I^2^ = 0%, SMD: 2.71, 95% CI [1.31, 4.12], p < 0.0001. GPX4‐1d: I^2^ = 91.1%, MD: 3.10, 95% CI [0.53, 5.66], ***p < 0.0001. GPX4‐3d: I^2^ = 84.5%, SMD: 3.55, 95% CI [2.34, 4.77], ***p < 0.0001. GPX4‐7d: I^2^ = 88.4%, MD: 2.58, 95% CI [1.34, 3.81], ***p < 0.0001. GPX4‐14d: I^2^ = 0%, SMD: 2.74, 95% CI [2.09, 3.39], ***p < 0.0001.

**FIGURE 8:**
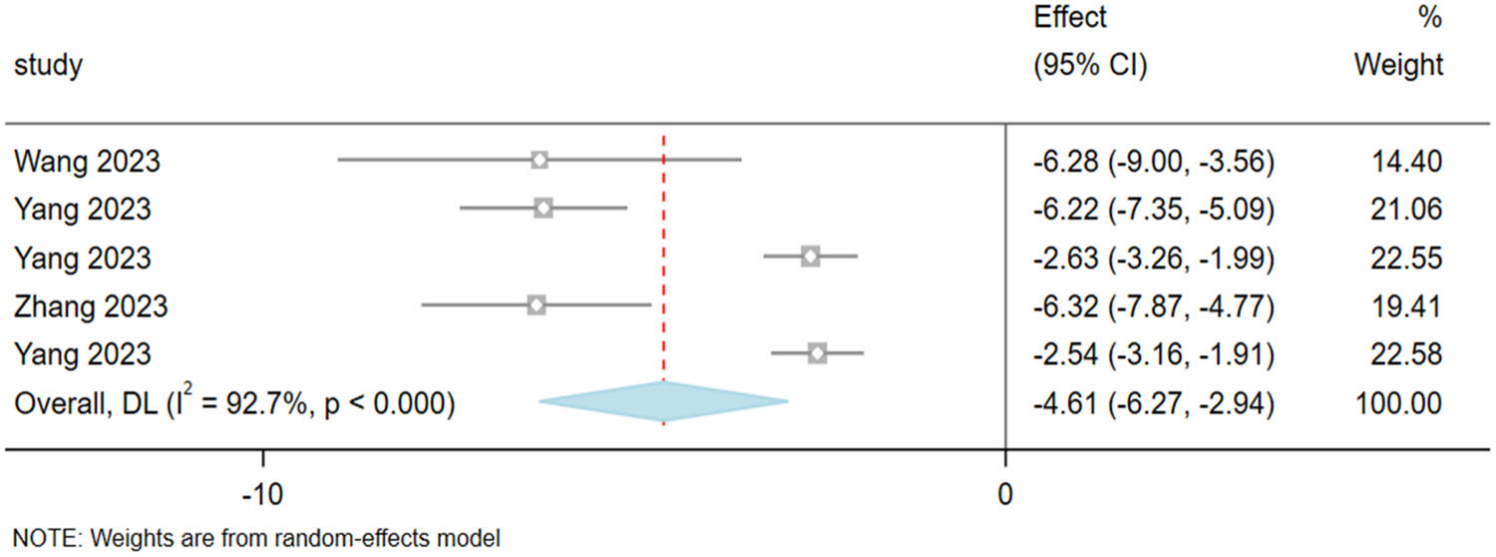
The result of ACSL4 based on acupuncture therapy in experimental cerebral stroke. Note: The ACSL4 levels in the acupuncture group were significantly reduced.

The meta‐analysis of five studies involving 270 rats evaluated the ferroptosis marker ACSL4 in brain tissue post‐stroke. The results (Figure 8) show that, compared to the model group, acupuncture significantly reduces the levels of ACSL4 in brain tissue after stroke, thereby inhibiting ferroptosis. ACSL4: I^2^ = 92.7%, high heterogeneity (I^2^ > 50%), random‐effects model, SMD: ‐4.61, 95% CI [‐6.27, ‐2.94], ***p < 0.0001. ACSL4 levels in the brain tissue of the acupuncture group were significantly reduced.

### 2.5 Subgroup Analyses and Sensitivity Analyses

To explore factors affecting result measurements, we stratified the included studies based on variables with high heterogeneity (I^2^ > 50%), as shown in Table s2. Based on the overall effects of acupuncture on improving neurological function and inhibiting ferroptosis, we conducted subgroup analysis and sensitivity analysis on lipid peroxidation index MDA, antioxidant index GSH, GPX4, and iron deposition index, respectively. Since other indicators showed no significant heterogeneity, additional subgroup and sensitivity analyses were not conducted. (1) The stratified analysis of MDA showed significant correlations of heterogeneity with treatment methods (p = 0.000). It was not related to animal species (p = 0.989), animal weight (p = 0.069), stroke model type (p = 0.545), or treatment time (p = 0.185). (2) The stratified analysis of GSH showed significant correlations between heterogeneity and treatment methods (p = 0.003), animal species (p = 0.012), animal weight (***p < 0.000), stroke model type (p = 0.035), and treatment time (p = 0.003). (3)The stratified analysis of GPX4 showed significant correlations between heterogeneity and treatment methods (p = 0.015), and animal weight (***p < 0.000). It was not related to animal species (p = 0.229), stroke model type (p = 0.818), or treatment time (p = 0.015). (4) The stratified analysis of iron content showed significant correlations between heterogeneity and animal species (p = 0.002) and animal weight (***p < 0.002). It was not related to treatment method (p = 0.788), stroke model type (p = 0.224), or treatment time (p = 0.183). Sensitivity analysis showed no substantial change in results after deleting any experiment.

### 2.6 Assessment of Publication Bias

**FIGURE 9:**
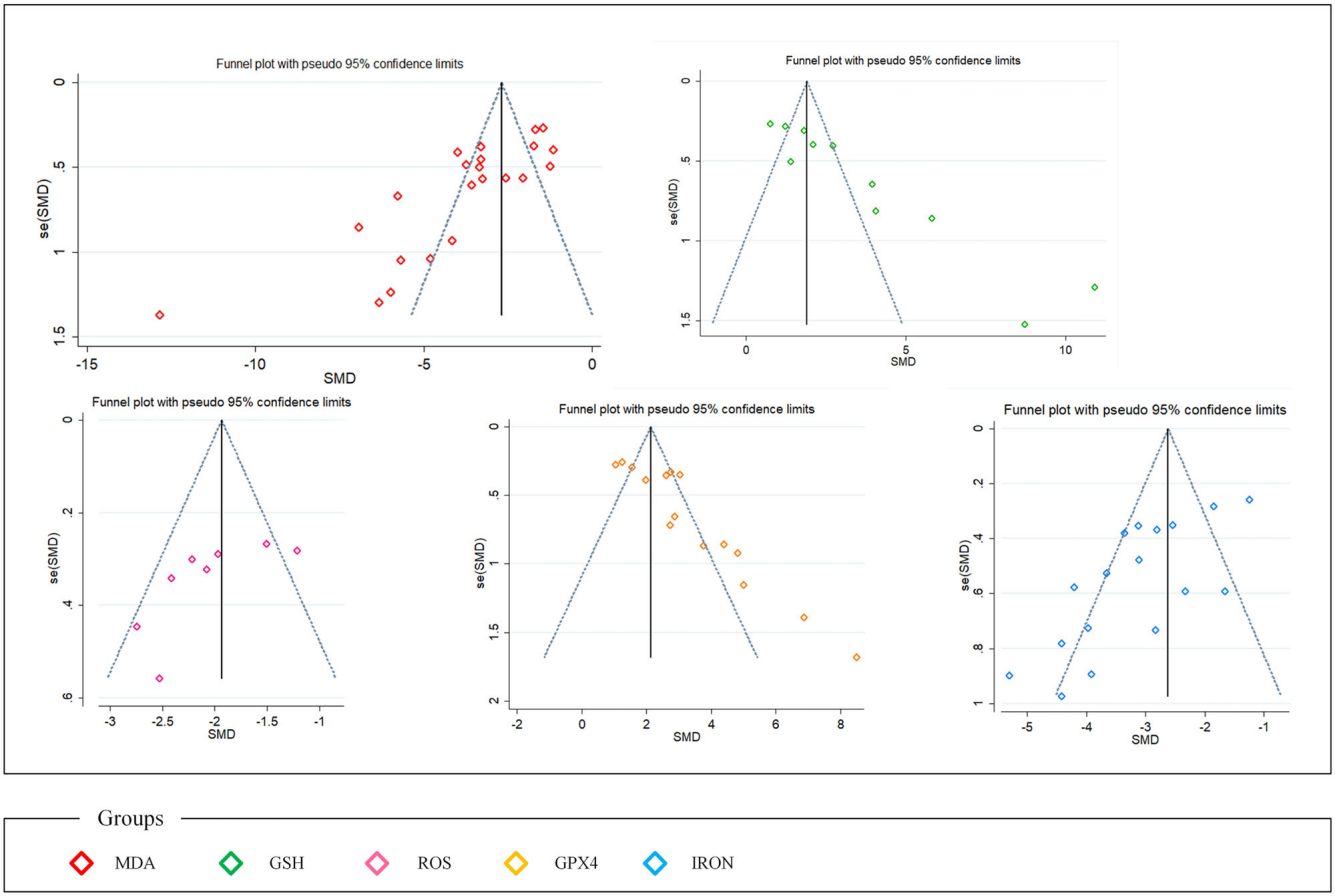
The funnel plot with MDA, GSH, ROS, and iron. Note: The red squares represent the funnel plot for MDA, the green squares represent the funnel plot for GSH, the pink squares represent the funnel plot for ROS, the yellow squares represent the funnel plot for GPX4, and the blue squares represent the funnel plot for IRON.

The funnel plot (Figure 9) shows asymmetry in some outcome measures, indicating potential publication bias. Egger's test detected publication bias in MDA, GSH, GPX4, and iron data (all **p < 0.01), but not in ROS (p = 0.071) or other outcomes (Table 2).

**TABLE 2:** Sensitivity analysis of all indexes.

Index	*t*‐values	*p*‐values	Index	*t*‐values	*p*‐values
MDA^**^	−5.51	0.000	Garcia	−1.40	0.395
GSH^**^	7.24	0.000	Longa	0.31	0.778
GPX4^**^	12.58	0.000	Longa (mNss)	−2.36	0.050
iron^**^	−4.01	0.001	FTH1	−2.48	0.089
ROS	−2.44	0.071	SOD	1.64	0.199
ACSL4	−2.60	0.080	TFR1	−2.81	0.107
BDNF	1.21	0.281	Zausinger	2.84	0.220
Ludmila Belayev	−0.08	0.938			

NOTE: **p < 0.01.

To further assess the robustness of the pooled results and potential publication bias, a trim‐and‐fill analysis was conducted for the four outcomes showing significant Egger's test results (MDA, GSH, GPX4, and iron). As shown in Figure 11, while the adjusted effect sizes were slightly reduced in magnitude compared to the original estimates, they remained statistically significant. This suggests that although publication bias may exist, it does not fully account for the observed treatment effects of acupuncture on ferroptosis‐related markers. Therefore, the overall conclusions remain robust (Table S4 and Figure 11).

## 3 Discussion

### 3.1 Efficacy of Acupuncture

**FIGURE 10:**
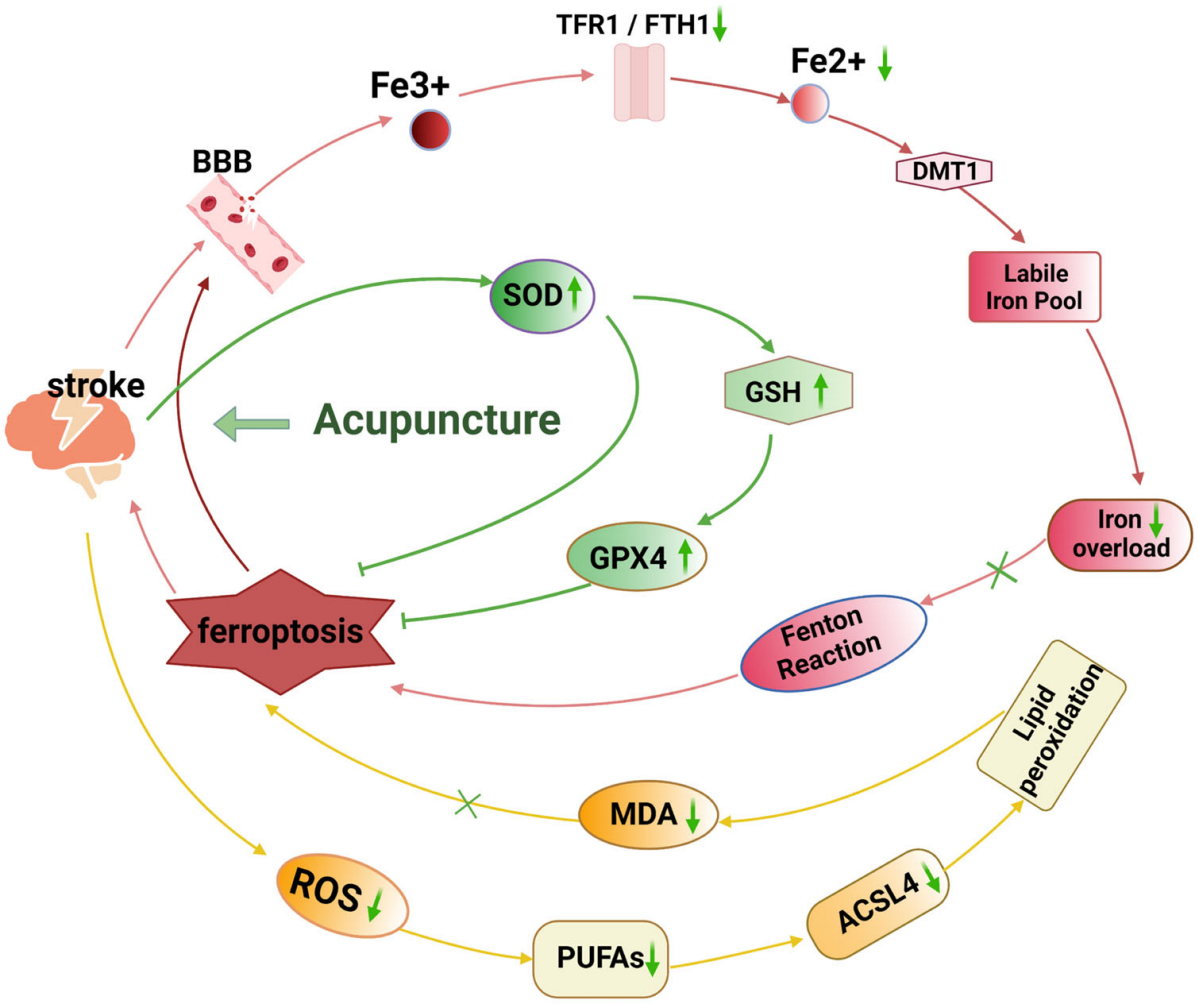
Mechanisms targeting ferroptosis inhibition in brain tissue through acupuncture treatment for experimental stroke. Note: The red pathway represents the iron metabolism mechanism, the yellow pathway signifies the lipid peroxidation mechanism, and the green pathway denotes the antioxidant mechanism. The green arrows and symbols highlight the markers of acupuncture targets.

This systematic review and meta‐analysis demonstrate that acupuncture may enhance neurological function and inhibit ferroptosis in experimental stroke animal models. The mechanisms involve the regulation of iron overload and metabolism (iron, TFR1, FTH1), reduction of lipid peroxidation (ROS, MDA), enhancement of antioxidant capacity in the animal model's brain tissue (GSH, GPX4, SOD), and the promotion of survival, regeneration, and maintenance of damaged neurons in the brain (BDNF). This work further identifies key acupuncture targets for inhibiting ferroptosis in brain tissue after stroke, as illustrated by the green arrows (**Figure** **10** ).

**FIGURE 11:**
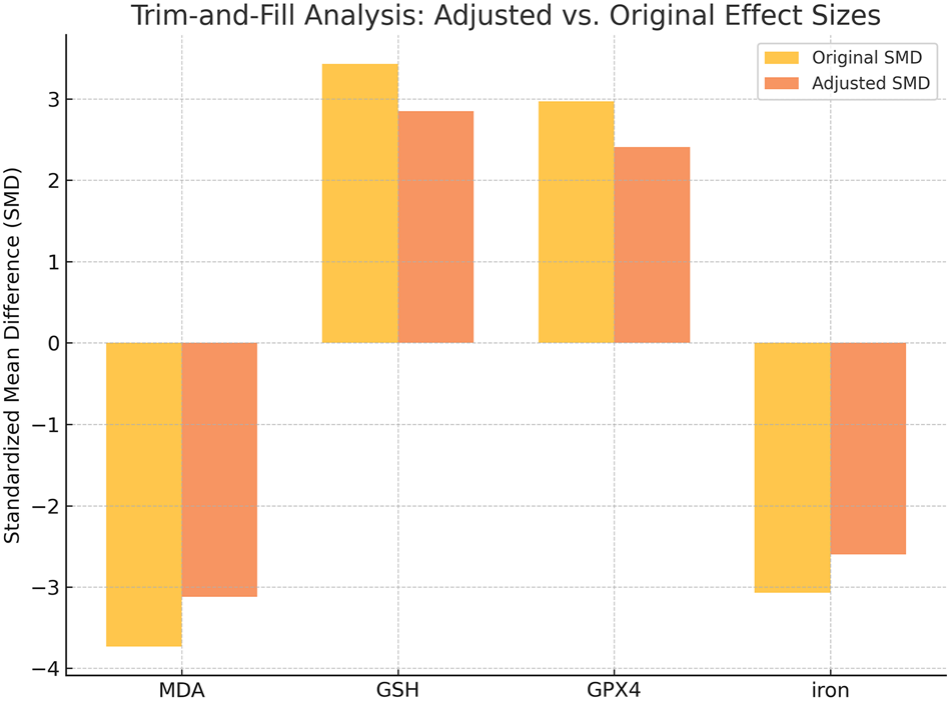
Trim‐and‐fill analysis. NOTE: Comparison of original and adjusted standardized mean differences (SMD) for MDA, GSH, GPX4, and iron. The yellow bars represent the originally pooled effect sizes from the meta‐analysis, while the orange bars represent the adjusted effect sizes calculated using the trim‐and‐fill method. This method accounts for possible publication bias by estimating the impact of potentially missing studies.

### 3.2 Heterogeneity Interpretation

The meta‐analysis revealed a certain degree of heterogeneity in brain tissue MDA, GSH, GPX4, and iron results. Subgroup analysis showed that heterogeneity was significantly associated with treatment methods, animal species, animal weight, and model selection following a stroke. We systematically reviewed all included studies to assess potential sources of heterogeneity. Further analysis suggested that variations in treatment methods were a primary contributor to heterogeneity. Significant variations in acupuncture methods and parameters (e.g., frequency, current density, acupoint selection) across studies may have influenced acupuncture efficacy. Additionally, the type and dose of anesthetics used during surgery may impact the experimental animals' condition and the effects of acupuncture, thus contributing to the heterogeneity of the results. However, studies using neuroprotective anesthetics were excluded during the selection process. Variations in animal species and weight also contributed to heterogeneity, though their impact was likely minimal. Standardizing animal models and strictly controlling experimental conditions are also feasible ways to reduce heterogeneity. Stroke model selection is another key determinant of heterogeneity. Ischemic and hemorrhagic strokes may differ in pathological mechanisms and treatment responses, so the chosen stroke model (MCAO or ICH) could affect the results. However, given the limited experimental evidence on acupuncture's role in ferroptosis post‐stroke, further studies are needed to strengthen these findings as more relevant evidence becomes available. Thus, these findings should be interpreted with caution.

### 3.3 Potential Mechanism

#### 3.3.1 Inhibition of Lipid Peroxidation

The imbalance between oxidative damage and antioxidant defense is central to ferroptosis, with ROS‐induced lipid peroxidation and redox imbalance being key factors in the occurrence and regulation of ferroptosis (Park and Chung 2019; Stockwell 2022). ROS are major inducers of lipid peroxidation (Xie et al. 2021). During a stroke, the disruption of the BBB allows iron and ferritin to enter the brain parenchyma, where they convert hydrogen peroxide (H_2_O_2_) into hydroxyl radicals (OH•) via the Fenton reaction, significantly increasing ROS production (Liu et al. 2024; Fan et al. 2024). ROS can attack polyunsaturated fatty acids (PUFAs) in cell membranes, triggering lipid peroxidation reactions that compromise membrane integrity and lead to ferroptosis. However, reducing ROS production after stroke not only protects membrane lipids from damage but also reduces oxidative damage to DNA and proteins, mitigating cell dysfunction and death, thereby preserving neurological function after stroke. MDA is one of the final products of lipid peroxidation. The accumulation of MDA signifies increased lipid peroxidation and represents the advancement of ferroptosis (Zhang et al. 2023). It can also react with intracellular proteins and DNA to form cross‐linked products, further damaging neuronal cell structure and function, exacerbating neurological deficits after stroke (Liu et al. 2022). ACSL4 is a key enzyme in lipid metabolism, playing a crucial role in ferroptosis (Liao et al. 2022; Qi et al. 2023). It is involved in the synthesis and remodeling of phosphatidylethanolamine (PE), particularly catalyzing the binding of PUFAs, such as arachidonic acid (AA), with CoA to form acyl‐CoA (Ding et al. 2023; Gan 2022). Post‐stroke, the expression of ACSL4 significantly increases, promoting the acylation of PUFAs and the formation of phospholipid hydroperoxides (PLOOH), thereby exacerbating lipid peroxidation and ferroptosis (Cui et al. 2021; Tuo et al. 2022). Evidence shows that inhibiting ACSL4 activity and reducing MDA levels in the MCAO rat model can decrease the generation of lipid peroxides, alleviating neural damage induced by ischemic stroke (Hu et al. 2024).

Our meta‐analysis supports these mechanisms, suggesting that acupuncture reduces ROS production in post‐stroke brain tissue, downregulates MDA and ACSL4 levels, inhibits lipid peroxidation, and may interrupt key ferroptosis pathways. This neuroprotective effect promotes the recovery of neurological function in brain tissue after stroke.

#### 3.3.2 Inhibition of Iron Overload and Regulation of Iron Metabolism

Iron homeostasis is crucial for normal brain physiology. During the pathological process of stroke, the balance of iron metabolism is disrupted, leading to iron overload, which is a primary cause of ferroptosis (Long et al. 2023). Iron overload following a stroke is a result of multiple factors. The breakdown of the BBB permits excessive iron to enter brain tissue, triggering iron overload (Chen et al. 2022). Transferrin (Tf) binds to TFR1 (Sanguigno et al. 2023), increasing intracellular iron levels, and the upregulation of TFR1 indirectly leads to the upregulation of FTH1 (Shi et al. 2024). Ferroportin (FPN) regulates intracellular iron balance by exporting iron, while ferritin (FT) reduces free iron levels by storing iron. These key steps in iron metabolism become imbalanced after a stroke, resulting in the accumulation of intracellular iron, catalyzing oxidative stress and lipid peroxidation, and ultimately leading to ferroptosis (Sun et al. 2022).

TFR1 is an important cell membrane protein primarily responsible for mediating the binding and uptake of Tf and iron (Sanguigno et al. 2023). Studies have shown that upregulation of TFR1 is closely associated with neuronal damage (Luo et al. 2023). Following cerebral ischemia‐reperfusion, increased TFR1 expression leads to iron overload and oxidative stress, further inducing neuronal ferroptosis (Yang et al. 2024). The occurrence of ferroptosis not only exacerbates acute neurological damage during stroke but may also result in chronic neurological dysfunction during the recovery phase, affecting long‐term prognosis. FTH1 plays a critical role in iron storage and detoxification by oxidizing highly reactive ferrous iron (Fe^2^⁺) to ferric iron (Fe^3^⁺) and safely storing it within the ferritin core, preventing oxidative stress and cellular damage caused by intracellular iron (Fang et al. 2021). However, FTH1 can also increase iron levels through autophagic degradation, which may promote ferroptosis (Chen et al. 2021).

The mechanisms of iron metabolism in brain tissue post‐stroke are complex. Our meta‐analysis indicates that acupuncture reduces post‐stroke iron accumulation in brain tissue, downregulates TFR1 and FTH1 levels, inhibits iron overload, and modulates iron metabolism. This modulation may influence the initiating factors of ferroptosis post‐stroke, ultimately protecting neuronal cells from death and promoting the recovery of neurological function.

#### 3.3.3 Enhancement of Brain Antioxidant Capacity

Following a stroke, the antioxidant defense system plays a crucial role in regulating ferroptosis, with GSH and GPX4 being key antioxidants and regulatory factors (Xu et al. 2023). GSH, a tripeptide composed of glutamate, glycine, and cysteine, protects cells from oxidative damage by scavenging free radicals and peroxides (Wu et al. 2004). GPX4 is the only antioxidant enzyme that converts GSH into GSSG while simultaneously transforming cytotoxic lipid hydroperoxides into non‐toxic lipid alcohols. The levels and activity of GPX4 are critical regulatory targets in ferroptosis, and its protective effects are essential for mitigating stroke‐induced neurological damage (Alim et al. 2019). Our meta‐analysis results indicate that acupuncture can enhance the antioxidant capacity of brain tissue after stroke by increasing the levels of GSH, GPX4, and SOD, thereby inhibiting iron overload. This modulation may counteract the factors leading to ferroptosis after stroke, thus protecting neuronal cells and promoting the recovery of neurological function. SOD is a crucial antioxidant enzyme present in nearly all aerobic organisms. It exerts significant protective effects in ferroptosis by scavenging superoxide anions, regulating hydrogen peroxide levels, reducing oxidative stress, and maintaining mitochondrial function (Yan et al. 2021). Multiple studies have demonstrated that the imbalance of the antioxidant system, which induces ferroptosis during stroke, is a key mechanism of neurological damage (Doll et al. 2019; von Mässenhausen et al. 2022; Xu et al. 2023; Yang and Stockwell 2016; Ye et al. 2022). Our findings suggest that acupuncture may regulate ferroptosis, mitigate stroke‐induced neuronal damage, and enhance neurological recovery in stroke animal models by modulating key antioxidants such as GSH, GPX4, and SOD.

#### 3.3.4 Promotion of Neuronal Survival and Regeneration

Neuronal cells in the brain are the fundamental units of the nervous system. On the one hand, in ischemic stroke, occlusion of cerebral blood vessels leads to a rapid depletion of glucose and oxygen in local brain tissue, which is the primary cause of neuronal damage. Intravenous thrombolysis can save the ischemic penumbra in a short period, but subsequent reperfusion introduces a heavier burden of ROS and inflammatory responses to the affected area (Hou and Brenner, 2024). On the other hand, in hemorrhagic stroke, the rupture of blood vessels causes irreversible mechanical damage to the brain, and the ensuing hematoma expansion, focal inflammation, ionic metabolic disorders, and accumulation of toxic substances further accelerate neuronal cell death (Han et al. 2024). Our meta‐analysis results indicate that acupuncture significantly increases BDNF levels in brain tissue after stroke. BDNF is a neurotrophic factor widely distributed in the central nervous system (Sun et al. 2023). Evidence shows that BDNF plays a critical role in promoting the survival, functional maintenance, and synaptic plasticity of neurons following stroke (Sayyah et al. 2022). Additionally, BDNF binds to its high‐affinity tyrosine kinase receptor B (receptor TrkB), activating multiple downstream signaling pathways to promote neuronal survival and growth, thereby supporting neuronal function (Kimura et al. 2016; Pandya et al. 2013). Although ferroptosis, as an atypical form of cell death, has a devastating impact on neurons, its adjustability provides researchers with the possibility of intervening in cell death programs. Our meta‐analysis confirms that ferroptosis likely contributes to neuronal cell death post‐stroke, involving complex mechanisms such as iron overload, lipid peroxidation, and antioxidant system dysregulation, along with multiple interacting signaling pathways. However, our findings suggest that acupuncture may target the key regulatory factor BDNF in brain tissue repair following stroke and inhibit the three main pathways of neuronal ferroptosis, thereby comprehensively modulating the recovery of neurological function after stroke.

#### 3.3.5 Regulation of the Nrf2–Keap1–GPX4 Pathway

Emerging evidence highlights the nuclear factor erythroid 2–related factor 2 (Nrf2)–Kelch‐like ECH‐associated protein 1 (Keap1)–GPX4 signaling pathway as a key modulator of ferroptosis. Under oxidative stress, Nrf2 dissociates from Keap1 and translocates into the nucleus, where it upregulates the expression of antioxidant genes, including GPX4 and heme oxygenase‐1 (HO‐1), thereby attenuating lipid peroxidation and ferroptotic cell death (Zhang et al. 2023; Dodson et al. 2019). Several experimental studies have demonstrated that acupuncture may activate this signaling cascade. For instance, electroacupuncture (EA) stimulation was shown to enhance Nrf2 nuclear translocation, upregulate GPX4 and HO‐1 expression, and reduce ROS and MDA levels in stroke models (Wang et al. 2015). These findings suggest that acupuncture may exert its anti‐ferroptotic effects by regulating redox homeostasis through the Nrf2–Keap1–GPX4 axis. Therefore, targeting this pathway may provide a promising mechanism through which acupuncture protects neural tissue and promotes recovery after stroke.

### 3.4 Influence of Acupuncture Parameters on Therapeutic Outcomes

In this systematic review and meta‐analysis, the included studies employed a variety of acupuncture methods and parameters, including EA, MA, and Moxi (Table s1). Commonly utilized acupoints encompassed DU26, GB7, and PC6, among others, which are traditionally associated with enhancing cerebral circulation, regulating Qi, and promoting overall neurological recovery. The selection of these acupoints is grounded in traditional Chinese medicine (TCM) principles, aiming to harmonize the flow of Qi and restore balance within the body (Table 4).

Regarding stimulation parameters (Table s1), EA frequencies ranged from low (2 Hz) to high (10 Hz), with current intensities typically maintained between 1 and 3 mA, tailored to the tolerance levels of the animal models. MA varied in terms of needle manipulation techniques, including twisting and lifting‐thrusting motions, applied at moderate intensities to prevent excessive stress or discomfort to the animals. Treatment durations were generally standardized, with most studies administering acupuncture sessions lasting approximately 20 min per session, conducted once or twice daily over a period of one to two weeks.

The heterogeneity in acupuncture parameters across studies may significantly influence the observed effects on ferroptosis inhibition and neurological function improvement. For instance, low‐frequency EA (e.g., 2 Hz) has been associated with promoting neurogenesis and anti‐inflammatory effects, whereas high‐frequency EA (e.g., 10 Hz) may be more effective in modulating autonomic nervous system activity and reducing oxidative stress. (Huang et al. 2025; Liu et al. 2025) Additionally, the combination of specific acupoints can engage multiple physiological pathways, potentially enhancing the overall therapeutic efficacy through synergistic mechanisms (Table 4).

However, the variability in acupuncture parameters poses challenges for the comparability and reproducibility of results. To address this, future research should strive towards standardizing acupuncture protocols or meticulously documenting all relevant parameters to facilitate replication and meta‐analytic integration. Establishing standardized operating procedures (SOPs) for acupuncture interventions, including precise acupoint selection, stimulation frequencies, intensities, and treatment durations, will enhance the reliability of preclinical studies and support their translation into clinical applications (Chen et al. 2024).

Moreover, understanding the dose‐response relationship of acupuncture parameters is crucial for optimizing therapeutic outcomes (Luo et al. 2024). Clinical trials should consider incorporating various acupuncture parameter settings to determine the most effective combinations for inhibiting ferroptosis and enhancing neurological recovery in stroke patients. By bridging the gap between animal model findings and clinical practice through standardized and well‐documented acupuncture protocols, the translational potential of acupuncture as a complementary therapy for stroke can be significantly advanced.

### 3.5 Critical Evaluation of Animal Models

The selection of appropriate animal models is paramount for elucidating the mechanisms underlying ferroptosis and evaluating the therapeutic efficacy of acupuncture in cerebral stroke. Among the included studies, the MCAO model was predominantly utilized, accounting for approximately 52.2% of the studies. The MCAO model is highly regarded for its ability to mimic ischemic stroke, replicating the occlusion and subsequent reperfusion that occurs in human ischemic events (Longa et al. 1989). This model facilitates the study of localized neuronal injury and the associated ferroptotic pathways, making it particularly relevant for assessing acupuncture's neuroprotective effects (Chen et al. 2025). However, variability in surgical techniques and the potential for collateral circulation can introduce inconsistencies across studies, potentially affecting the reproducibility of results. In contrast, the ICH model, employed in 21.7% of the studies, accurately represents hemorrhagic stroke, allowing for the investigation of blood‐induced oxidative stress and iron overload, key components of ferroptosis (Chiang et al. 2025). While this model provides valuable insights into the distinct pathophysiological processes of hemorrhagic strokes, its complexity and the variability in hemorrhage size and location pose challenges for standardization and reproducibility.

The MCAO/R model integrates reperfusion injury, a critical aspect of ischemic stroke management in clinical settings (Liu et al. 2025). Approximately 8.7% of the studies utilized this model to explore the dual impact of ischemia and reperfusion on ferroptosis and neurological outcomes. The inclusion of reperfusion introduces additional variables, such as inflammatory responses and oxidative stress, which may amplify the ferroptotic processes and influence the efficacy of acupuncture interventions (Jian et al. 2024). However, the increased complexity of this model necessitates meticulous surgical precision to minimize variability.

Less commonly used, the MID model and the 4‐VO model each offer unique advantages for studying chronic and global cerebral ischemia, respectively (Kaneko et al. 1985). The MID model, representing chronic ischemic conditions, is instrumental for examining long‐term neurological deficits and the sustained impact of acupuncture on ferroptosis (Chen et al. 1994). Conversely, the 4‐VO model, while effective for inducing widespread cerebral ischemia, is highly invasive and may result in higher mortality rates, limiting its applicability for longitudinal studies (Pulsinelli and Brierley 1979).

The PSD model, though utilized in a smaller fraction of studies (4.3%), integrates behavioral assessments with neurological injury, providing a comprehensive framework for evaluating the multifaceted effects of acupuncture (Willner et al. 1987). This model is particularly useful for exploring the interplay between ferroptosis and psychological outcomes post‐stroke. However, accurately modeling depression in animals remains challenging due to the subjective nature of behavioral assessments (Willner 1990).

In summary, while the diversity of animal models enhances the breadth of ferroptosis research in stroke, it also introduces heterogeneity that can complicate the synthesis and interpretation of results. Future studies should strive for greater standardization in model selection and procedural methodologies to improve comparability. Additionally, adopting a combination of models may provide a more holistic understanding of acupuncture's mechanisms in mitigating ferroptosis and promoting neurological recovery. Tailoring acupuncture protocols to the specific characteristics of each animal model will further refine the translational potential of preclinical findings to clinical applications.

### 3.6 Clinical Relevance and Potential Translational Implications

The present meta‐analysis highlights the beneficial effects of acupuncture on ferroptosis inhibition and neurological function improvement in various stroke animal models. Although these findings are based on experimental research, they suggest important clinical implications. Firstly, the regulation of ferroptosis—a form of iron‐dependent cell death—opens a new therapeutic avenue for stroke management (Liu et al. 2025). To be frank, our research team initially focused on investigating the efficacy and safety of acupuncture in promoting post‐stroke angiogenesis and neuroregeneration (Zhang et al. 2023). However, through extensive literature review and reflections on clinical practice, we have come to recognize ferroptosis as a significant therapeutic target (Ge et al. 2024). Stroke is closely associated with iron homeostasis and lipid metabolism balance, with disruptions in these processes being key and indispensable pathological factors contributing to ferroptosis (Kalaria and Englund 2025). This realization has reinforced the necessity of studying this mechanism further. Targeting ferroptosis through acupuncture could complement current interventions that focus on angiogenesis and neuroprotection, potentially reducing secondary brain injury (Yang et al. 2025).

Secondly, as cerebral stroke remains a leading cause of mortality and disability worldwide, integrating acupuncture into standard care protocols might offer a cost‐effective and culturally acceptable adjunct therapy, especially in regions with a strong tradition of acupuncture use.

Despite promising animal data, the translation to clinical practice requires rigorously designed clinical trials. Future randomized controlled trials (RCTs) could measure not only standard functional outcomes (e.g., National Institutes of Health Stroke Scale [NIHSS] or modified Rankin Scale[mRS]) but also biomarkers linked to ferroptosis (e.g., serum or imaging markers of lipid peroxidation and iron metabolism) (Wei et al. 2025). Such studies would help clarify whether acupuncture‐mediated ferroptosis inhibition directly correlates with improved long‐term neurological recovery in patients with ischemic or hemorrhagic stroke. Additionally, standardized acupuncture protocols (e.g., specific acupoints, stimulation frequency, intensity, and duration) need to be established to ensure reproducible and clinically meaningful outcomes. These protocols should be informed by the parameters found effective in animal models, with appropriate modifications for patient safety and comfort.

Finally, combining acupuncture with conventional stroke therapies—such as thrombolysis, endovascular treatment, and neuroprotective agents—could potentially yield synergistic benefits (Liu et al. 2025). Further research is warranted to determine optimal treatment windows and whether certain subpopulations of stroke patients (e.g., those with massive infarction or comorbidities) might respond more favorably to acupuncture. By leveraging the emerging understanding of ferroptosis and integrating evidence‐based acupuncture protocols, future translational studies have the potential to significantly improve stroke outcomes and reduce the global burden of this disease.

### 3.7 Assessment of Publication Bias

Despite the comprehensive nature of this meta‐analysis, our findings indicate the presence of publication bias in several key outcome measures, including MDA, GSH, GPX4, and iron levels, as evidenced by funnel plot (Figure 9) asymmetry and significant results from Egger's tests (all p < 0.01) (Table 2). Publication bias occurs when the likelihood of a study being published is influenced by the trends of its results, often leading to an overrepresentation of studies with positive findings (Hohlfeld et al. 2024). In the context of our analysis, this bias may result in an inflated estimation of acupuncture's efficacy in inhibiting ferroptosis and improving neurological outcomes post‐stroke, as studies with null or negative results might remain unpublished or inaccessible.

The detection of publication bias undermines the generalizability of our conclusions, as the pooled effect sizes may not accurately reflect the true impact of acupuncture across all conducted research (Egger et al. 1997). This limitation highlights the need for cautious interpretation of the positive associations observed in this meta‐analysis. To mitigate the influence of publication bias, future research should adopt several strategies. Firstly, pre‐registering studies and clinical trials in public databases can promote transparency and reduce selective reporting. Additionally, encouraging the publication of negative or inconclusive findings is crucial for providing a balanced evidence base (Schweinfurth and Frommen 2025).

Furthermore, employing advanced statistical techniques, such as trim‐and‐fill analysis or selection models, in future meta‐analyses can help adjust for potential publication bias and yield more accurate effect estimates (Xie et al. 2025). By addressing publication bias through these methodological enhancements, subsequent meta‐analyses can better represent the true efficacy of acupuncture in modulating ferroptosis and improving neurological function in cerebral stroke models. Ultimately, acknowledging and addressing publication bias is essential for advancing the scientific rigor and clinical relevance of acupuncture research in stroke therapy (Paturu et al. 2024).

### 3.8 Limitations

Although all studies indicate that acupuncture inhibits ferroptosis‐related factors and improves neurological function after stroke, our analysis revealed publication bias through funnel plot asymmetry (Figure 9) and Egger's test (Table 2). This bias may arise from unpublished non‐significant findings, with negative results less frequently appearing in the literature. Publication bias is common in medical research, particularly in small‐scale clinical trials. Researchers and journals tend to favor significant positive results while paying less attention to negative or non‐significant findings. This selective reporting can skew the outcomes of systematic reviews and meta‐analyses toward positive effects. Furthermore, small‐scale studies, due to design flaws or the non‐publication of contrary results, may overestimate treatment effects. These studies often face methodological limitations, such as insufficient sample sizes, inadequate randomization, and blinding procedures. Such factors can lead to biased effect estimates, potentially exaggerating the treatment efficacy. Therefore, despite the current evidence suggesting that acupuncture has potential in improving ferroptosis and neurological function after stroke, these findings should be interpreted with caution.

## 4 Materials and Methods

This study aimed to investigate the inhibitory effects of acupuncture on ferroptosis following stroke and its protective role in neurological function. The design and implementation of this systematic review and meta‐analysis followed the guidelines of the preferred reporting items for systematic reviews and meta‐analyses (PRISMA) (Page et al. 2021). We have registered this meta‐analysis with PROSPERO (CRD42024569661).

### 4.1 Search Strategy and Study Selection

A comprehensive literature search was conducted from the inception of the databases up to May 30, 2024, across both English and Chinese databases, including Web of Science, Embase, Ebsco, PubMed, Cochrane library, and Chinese databases such as China biomedical literature database (CBM), CNKI, Wanfang database (WF), and VIP database for Chinese technical periodicals. This inclusion aimed to minimize language bias and ensure a more comprehensive coverage of acupuncture‐related studies, which are frequently published in Chinese journals. The search strategy utilized a combination of free‐text terms and MeSH terms to construct the search keywords. Additionally, the reference lists of the retrieved articles were manually searched to identify any potential eligible studies. This extensive search aimed to maximize the retrieval of relevant studies and ensure a thorough analysis of the existing evidence base. The search strategy used in PubMed is illustrated in Table s3.

### 4.2 Inclusion and Exclusion Criteria

We included all identified studies on acupuncture in experimental stroke related to ferroptosis and neurological function. For neurological function assessment in animal models, outcome measures included Longa and/or Longa (mNSS) scores and/or Zausinger and/or Garcia and/or Ludmila Belayev scores and/or BDNF levels as indicators of brain tissue neuroregeneration and repair. For detecting ferroptosis in brain tissue, outcomes were categorized into three aspects: lipid peroxidation (measured by MDA and/or ROS), iron metabolism (measured by iron ions and/or FTH1 and/or TFR1), and antioxidation (measured by GPX4 and/or GSH) and/or SOD, including studies with specific ferroptosis marker ACSL4. Infarct volume was extracted as an outcome measure where appropriate.

Inclusion criteria were as follows: (1) studies on experimental ischemic stroke induced by vascular occlusion or rupture in animal models of stroke (including both ischemic and hemorrhagic stroke), (2) interventions involving acupuncture, including EA, MA, or Moxi, (3) control groups that did not receive acupuncture treatment (non‐EA or sham‐MA), (4) studies that included neurological function assessments, such as but not limited to Longa scores, Longa (mNSS) scores, Zausinger scores, Garcia scores, and Ludmila Belayev scores, as well as BDNF levels as markers of neuroregeneration and repair, (5) studies that used at least one of the following ferroptosis markers as an outcome measure: iron ions, FTH1, TFR1, MDA, ROS, GPX4, GSH, SOD, or ACSL4, and (6) studies providing clear inter‐group differences (p‐values) and reporting significance levels (e.g., *p* < 0.01 or *p* < 0.05).

Exclusion criteria were: (1) studies on diseases not caused by cerebral stroke, (2) studies involving non‐murine, or female murine models, (3) clinical studies, reviews, in vitro studies, in sillico studies, and conference reports, (4) studies lacking a control group or involving combined interventions of acupuncture with other therapies/drugs, (5) studies with unavailable effective data or inaccessible full text, (6) duplicate publications, with preference given to the most recent data in cases of duplicated data, and (7) studies with inappropriate outcome measures.

### 4.3 Data Extraction

Two researchers (ZWY and LDL) independently conducted literature searches based on the predetermined inclusion and exclusion criteria. Initially, titles and abstracts were screened to exclude irrelevant studies. Subsequently, the full texts of potentially eligible studies were thoroughly examined. For the included studies, the following information was extracted: (1) the name of the first author and year of publication; (2) animal information, including species, sex, weight, and sample size; (3) stroke model establishment; (4) treatment information, including the timing of initial treatment, type and method of treatment (acupoints, frequency, current, etc.), duration of treatment, and comparable treatment in the control group; and (5) outcome measurement information, including time points for each outcome measure, measurement methods, units of measurement, outcome data, and corresponding *p*‐values for outcome evaluation. If the results were presented graphically, we attempted to contact the authors to obtain the original data. If the authors did not respond, the graphical data were quantified using WebPlot Digitizer 4.5 software. When multiple time points were reported for the treatment group, data from each time point were extracted and recorded separately for meta‐analysis. If the reported data were in the format of standard error, conversion to standard deviation was performed using a defined formula. All extracted data were manually recorded by the two researchers (ZWY and LDL) independently in a tabular database. Any discrepancies during the extraction process were resolved through consultation with a third researcher (ZXZ).

### 4.4 Quality Assessment

Two authors independently assessed the methodological quality of the included studies using the collaborative approach to meta‐analysis and review of animal data from experimental studies (CAMARADES) 10‐item checklist (Macleod et al. 2004): (1) peer‐reviewed publication, (2) statement of temperature control, (3) random allocation to treatment or control group, (4) blinded induction of model (5) blinded assessment of outcomes, (6) use of anesthetic without significant intrinsic neuroprotective activity, (7) appropriate animal model, (8) compliance with animal welfare regulations, (9) statement of potential conflicts of interest, and (10) statement of funding. The total quality score for each study was 10 points, and the median score was calculated for the group. Discrepancies were resolved through discussion between the two authors (ZWY and LDL) or referred to an arbitrator (ZXZ).

### 4.5 Statistical Analysis

Statistical analyses were performed using STATA software version 16. The outcome measures in this study were continuous variables, varying in measurement methods and units across studies. To assess the overall effect size, we used standardized mean difference (SMD) and its corresponding 95% confidence interval (CI). Effect sizes were evaluated using Cohen's classification (Table 3) (Schober et al. 2021). A threshold of p < 0.05 was used to determine statistical significance. Heterogeneity of the data was assessed using the I‐squared (*I* ^2^) statistic. An *I* ^2^ value of less than 50% indicated insignificant statistical heterogeneity, in which case a fixed‐effects model was used. Conversely, if *I* ^2^ was greater than 50%, a random‐effects model was employed, and sensitivity analysis was conducted to explore potential sources of heterogeneity. Publication bias was assessed using funnel plots and the Egger test (Egger et al. 1997).

**TABLE 3:** The effect size represented by SMD.

Standardized mean difference (Cohen's d)	
Magnitude of the measure	Suggested interpretation
<0.10	Trivial effect
0.10‐0.34	Small effect
0.35‐0.64	Medium effect
0.65‐1.19	Large effect
≥ 1.20	Very large effect

**TABLE 4:** Acupoint selection for ferroptosis‐related stroke models: Meridian classification, functions, location and therapeutic applications.

**Meridian/** **category**	**Acupoint code and name**	**Characteristics** **/functions**	**Typical location**	**Stroke model application**	**Included studies**
**Du (Governor Vessel)**	DU26 (or GV26), Shuigou	Promotes resuscitation and restores consciousness; emergency revival acupoint.	At the junction of the upper and middle thirds of the philtrum on the midline of the upper lip.	MCAO MCAO / R	Wang 2023 Wu 2023 Zhang 2024
	DU20 (or (GV20), Baihui	Raises “Yang Qi”, improves cerebral circulation, calms the spirit; regulates brain function.	At the vertex of the head on the midline, approximately at the intersection of a line connecting the apexes of the ears.	MCAO MCAO/R	Chen 2022 Yang 2023 Gao 2023 Li 2022 Dai 2024
	DU14 (or GV14), Dazhui	Clears heat, expels wind, supports immune function.	Below the spinous process of the 7th cervical vertebra.	ICH MCAO/R	Li 2022 Wang 2024 Wu 2023
	DU16 (or GV16), Fengfu	Regulates brain function, supports consciousness, dispels “wind”.	At the nape of the neck, just below the occipital protuberance.	ICH	Dai 2024 Liang 2022
	DU4 (or GV4), Mingmen	Tonifies Kidney “Yang Qi”, strengthens vital energy.	On the midline of the lower back, approximately at the level of the L2 spinous process (near the "Mingmen").	MCAO/R 4‐VO	Zhang 2023 Li 2021 Zuo 2017
	DU23 (or GV23), Shangxing	Clears the mind, improves cognitive function, alleviates mental disturbances.	On the head, just above the anterior hairline (near the glabella).	MCAO/R MID	Zhang 2014 Zuo 2017 Liu 2005
**Gallbladder**	GB7, Qubin	Dispels “wind”, alleviates headache, supports temporal and visual circulation.	On the lateral aspect of the head/temple region.	ICH MCAO/R	Lin 2024 Lin 2015 Zhang 2023
**Pericardium**	PC6, Neiguan	Calms the mind, regulates heart “Qi”, alleviates nausea, and stabilizes autonomic function.	On the forearm, 2 cun proximal to the wrist crease, between the tendons of the palmaris longus and flexor carpi radialis.	MCAO/R	Liu 2005 Zhang 2024 Wang 2024
**Spleen**	SP6, Sanyinjiao	Nourishes blood, harmonizes Spleen, Liver, and Kidney, promotes circulation, and has calming effects.	On the medial aspect of the lower leg, approximately 3 cun above the medial malleolus, posterior to the medial border of the tibia.	MCAO / R ICH	Wang 2022 Wang 2024 Dai 2024
	SP10, Xuehai	Invigorates blood, resolves stasis, and clears heat.	On the thigh, about 2 cun above the patella, located on the bulge of the vastus medialis.	ICH	Wu 2023 Yang 2023
**Stomach**	ST36, Zusanli	Tonifies Qi and blood, enhances immune function, and supports digestion.	On the lower leg, approximately 3 cun below the lateral knee, 1 finger‐breadth lateral to the anterior crest of the tibia.	MCAO/R ICH MID	Gao 2023 Dai 2024 Lin 2015 Zhang 2014
	ST6, Jiache	Benefits the muscles, improves facial circulation, and relieves muscle tension.	On the face, at the prominence of the angle of the jaw.	MCAO / R	Li 2022 Wang 2023
**Conception vessel**	CV17, Dhanzhong	Regulates “Qi”, relieves chest congestion, and calms the mind.	On the anterior midline of the chest, at the level of the fourth intercostal space.	MCAO/R	Liang 2022 Zhang 2024
	CV12, Zhongwan	Strengthens the Spleen, harmonizes stomach function, and aids digestion.	On the midline of the abdomen, midway between the xiphoid process and the umbilicus.	MCAO/R MID	Zhang 2014 Lin 2015 Liu 2005
	CV6, Qihai	Tonifies original “Qi”, strengthens overall vitality, and supports systemic energy.	1.5 cun below the umbilicus on the anterior midline.	MID MCAO/R	Zuo 2017 Liu 2005 Lin 2024
	CV14, Jvque	Regulates “Qi”, strengthens heart function, and supports the flow of vital energy.	On the anterior midline of the chest, approximately 4 cun above the umbilicus.	MID MCAO/R	Li 2021 Lin 2024 Zhang 2014
**Large intestine**	LI11, Quchi	Clears heat, cools the blood, resolves dampness, and possesses anti‐inflammatory properties.	At the lateral end of the elbow crease, on the radial side of the tendon of the biceps brachii.	4–VO MCAO/R	Lin 2024 Wang 2022 Wu 2023
**Extra points**	EX‐HN3, Yintang	Calms the mind, alleviates headache, and reduces stress.	Located between the medial ends of the eyebrows.	MCAO/R ICH	Lin 2015 Zhang 2023 Wang 2023

### 4.6 Subgroup Analysis

We conducted subgroup analyses to explore potential sources of heterogeneity in the studies. Subgroups were defined based on various factors, including animal species, weight, model, acupuncture intervention timing, intervention methods, and intergroup differences. When significant heterogeneity was observed (*I* ^2^ > 50%), we further performed subgroup analyses and sensitivity analyses using Stata 16 software.

## 5 Conclusions

In conclusion, our findings indicate that acupuncture may inhibit ferroptosis and improve neurological function in adult animal stroke models. The potential mechanisms include: regulation of brain tissue iron overload and metabolism (iron, TFR1, FTH1); reduction of lipid peroxidation damage (ROS, MDA) and enhancement of antioxidant capacity (GSH, GPX4, SOD); and promotion of neuronal survival, regeneration, and functional maintenance (BDNF). Together, these effects contribute to the neurological function promotion of acupuncture.

## Author Contributions

**Wenyu Zhang**: conceptualization, software, data curation, investigation, validation, formal analysis, visualization, writing–original draft, methodology, writing–review and editing. **Xiaoxi Liu**: Conceptualization and methodological development Software, data curation, and validation Formal analysis, visualization, and investigation Critical review and editing of the manuscript. **Xuyang Feng**: conceptualization, methodology, software, data curation, validation, writing–original draft, writing–review and editing, investigation, visualization. **Donglei Lu**: conceptualization, investigation, methodology, software, data curation, formal analysis, writing–review and editing, validation, visualization, writing–original draft. **Ruiyu Li**: supervision, writing–review and editing, conceptualization, methodology, software, data curation, formal analysis, validation, investigation. **Haizhen Guo**: writing–review and editing, supervision, methodology, conceptualization, software, data curation, formal analysis, validation, investigation. **Xuezhu Zhang**: project administration, supervision, funding acquisition, resources, writing–review and editing. **Kun Nie**: Conceptualisation, methodology, software, investigation, validation, formal analysis, visualisation, manuscript draughting, and editing.

## Conflicts of Interest

The authors declare that the research was conducted in the absence of any commercial or financial relationships that could be construed as a potential conflict of interest.

## Peer Review

The peer review history for this article is available at https://publons.com/publon/10.1002/brb3.70507

## Supporting information




**Table s1** Characteristics of the 23 included studies.

**Table s2** Subgroup analyses for the effect of acupuncture on ferroptosis after stroke.

**Table s3** Search Strategy.

**Table s4** Trim‐And‐Fill Analysis Results.

## Data Availability

Data will be made available on request.
