# Supplementary material for: Experimental Evidence on Acupuncture Targeting Ferroptosis for Neurological Function Improvement in Cerebral Stroke: A Systematic Review and Meta‐Analysis
Source: Brain Behav. 2025 Aug 21;15(8):e70507. doi: 10.1002/brb3.70507 (PMC12370861; doi:10.1002/brb3.70507)
Supplement: Supplementary file 1 — Table s1 Characteristics of the 23 included studies. [file BRB3-15-e70507-s003.docx]

**Supplementary Table 1.** Characteristics of the 23 included studies.

| **Study**  **(years)** | **Species (Sex, n = experimental /control group )** | **Weight** | **Model (method)** | **Treatment group**  **(Method to acupuncture)** | **Control group** | **Outcome Index (time)** | **Intergroup Differences** | **Sampling location** |
| --- | --- | --- | --- | --- | --- | --- | --- | --- |
| Wang 2023  (53) | SD rats  （Male, 7 / 7） | 210 - 230 g | MCAO | EA 30 min after reperfusion per day, with frequency of 3.85 Hz / 6.25 Hz and  current density of 0.8~1.3 mA, (DU26, DU20). | Non - EA | 1. f (1d,2d,3d);  2. g (1, 3d);  3.i (3d);  4.o (3d);  5.q (3d); | 1. p＜0.01;  2. p＜0.01;  3. p＜0.01;  4. p＜0.01;  5. p＜0.01; | Ischemic brain tissue |
| Chen 2022  (40) | Wistar rats  (male, 12 / 12) | 300 ± 20 g | ICH | EA 30 min after surgery  per day, with frequency of 2Hz / 15 Hz and  current density of 2 mA, (DU20，DU14). | Non - EA | 1. b (3d,7d,14d);  2. h (3d, d,14d);  3. j (14d); | 1. p＜0.01;  2. p＜0.01;  3. p＜0.01; | hematoma brain tissue |
| Wu 2023  (37) | SD rats  （Male, 20 / 20） | 180 - 230 g | MCAO / R | EA 20 min after reperfusion  per day, with frequency of 2Hz / 15 Hz and  current density of 1-2 mA, (GV20, GV16, GV14). | Non - EA | 1. a (7d);  2. g (7d);  3. n (7d);  4. h (7d);  5. l (7d); | 1. p＜0.01;  2. p＜0.01;  3. p＜0.01;  4. p＜0.01;  5. p＜0.01; | Ischemic brain tissue |
| Li 2022  (47) | SD rats  (Male, 8 / 8） | 280 ± 10 g | ICH | SA 30 min after surgery  per day, with frequency of 180 ± 20 r/min, (DU20, GB7). | Non - SA | 1. c (6h, d,3d,7d);   2. o (6h, d,3d,7d);  3. i (6h, d,3d,7d);  4. l (6h, d,3d,7d);  5. h (6h, d,3d,7d); | 1. p＜0.01;  2. p＜0.01;  3. p＜0.01; | hematoma brain tissue |
| Zhang 2024  (52) | SD rats  (Male, 34 / 34） | 240 - 280 g | MCAO / R | MA 30 min after reperfusion per day, (PC6, GV26). | Non - MA | 1.f (1d);  2. g (1d);  3.i (1d);  4.h (1d);  5.l (1d); | 1. p＜0.05;  2. p＜0.01;  3. p＜0.01;  4. p＜0.01;  5. p＜0.01; | The ischemic area of the right cerebral cortex |
| Wang 2024  (43) | Wistar rats  (male, 10 / 10) | 150 - 200 g | MCAO / R | EA 30 min after reperfusion  per day, with frequency of 2Hz / 15 Hz and  current density of 1 mA, (GV26, SP6, PC6). | Non - EA | 1. d (1d,3d);  2. n (1d,3d);  3. l (1d,3d);  4. o (1d,3d);  5. j (1d,3d); | 1. p＜0.05;  2. p＜0.01;  3. p＜0.01;  4. p＜0.01;  5. p＜0.01; | The ischemic area of the left cerebral cortex |
| Dai 2024  (46) | SD rats  (Male, 15 / 15） | 200 - 220 g | ICH | MA 30 min after reperfusion per day, (GB7, GV20). | Non - MA | 1. c (1d,3d,7d);  2. o (1d,3d,7d);  3. l (1d,3d,7d);  4. n (1d,3d,7d); | 1. p＜0.05;  2. p＜0.05;  3. p＜0.05;  4. p＜0.05; | hematoma brain tissue |
| Yang 2023  (41) | SD rats  （Male, 36 / 36） | 200 - 220 g | MCAO / R | MA 30 min after reperfusion per day, (GV20). | Non - MA | 1. a (3d,7d,14d); 2. k (3d,7d,14d);   3. l (3d,7d,14d);  4. h (3d,7d,14d);  5. q (3d,7d,14d); | 1. p＜0.05;  2. p＜0.05;  3. p＜0.05;  4. p＜0.05;  5. p＜0.05; | Ischemic brain tissue |
| Gao 2023  (44) | SD rats  （Male, 10 / 10） | 240 ± 10 g | ICH | EA treatment begins on the second day after surgery, with frequency of 2Hz / 15 Hz and  current density of 2mA, (GV26, SP6,PC6). | Non - EA | 1.d (3d);  2. h (3d);  3.l (3d);  4.n (3d);  5.o (3d); | 1. p＜0.05;  2. p＜0.05;  3. p＜0.05;  4. p＜0.05;  5. p＜0.05; | hematoma brain tissue |
| Wang 2022  (48) | SD rats  (20 / 20） | 210 - 230 g | MCAO / R | MA 30 min after reperfusion per day, (DU20, GV26, GV14). | Non - MA | 1. e (3d);  2. g (3d);  3. k (3d);  4. l (3d);  5. h (3d); | 1. p＜0.05;  2. p＜0.01;  3. p＜0.01;  4. p＜0.01;  5. p＜0.01; | Ischemic hippocampus |
| Liang 2022  (42) | SD rats  （Male, 30 / 30） | 230 ± 20 g | MCAO / R | P-EA 14 days before surgery, with frequency of 2Hz / 15 Hz and  current density of 1mA, (GV20, ST6). | Non - p - EA | 1. b (1d,3d,7d);  2. g (3d);  3. h (1d,3d,7d);  4. n (1d,3d,7d);  5. o (1d,3d,7d);  6. ROS (1d,3d,7d); | 1. p＜0.05;  2. p＜0.05;  3. p＜0.05;  4. p＜0.05;  5. p＜0.05;  6. p＜0.05; | Ischemic brain tissue |
| Li 2021  (45) | SD rats  （Male, 24 / 24） | 280 ± 10 g | ICH | MA 30 min after surgery  per day, (GV20, GB7). | Non - MA | 1. c (1d,3d,7d);  2. l (1d,3d,7d); | 1. p＜0.05;  2. p＜0.05; | hematoma brain tissue |
| Zhang 2023  (49) | SD rats  （Male, 20 / 20） | 220 ± 10 g | MCAO/ R | Moxi 30 min after surgery | Non - Moxi | 1. e (7d);  2. g (7d);  3. l (7d);  4. o (7d);  5. n (7d);  6. q (7d);  7. h (7d); | 1. p＜0.01;  2. p＜0.01;  3. p＜0.01;  4. p＜0.05;  5. p＜0.01;  6. p＜0.01;  7. p＜0.01; | Ischemic brain tissue |
| Zhang 2014  (55) | Wistar rats  (male, 10 / 10) | 300 - 320 g | MID | MA 30 min after the completion of modeling per day, (CV17, CV12, CV6, SP10, ST36) | Sham - MA | 1. m | 1. p＜0.01 | brain tissue |
| Zuo 2017  (57) | SD rats  （Male, 24 / 24） | 200 - 250 g | 4 - VO | EA 20 min after reperfusion  per day, with frequency of 40Hz / 50 Hz (GV4, GV20, ST36) | Non - EA | 1. a (1d)  2. m (3d) | 1. p＜0.01  1. p＜0.01 | Carotid artery blood and hippocampus |
| Lin 2024  (54) | SD rats  （Male, 12 / 12） | 300 ± 20 g | MCAO / R | EA 30 min after reperfusion  per day, with frequency of 2Hz / 20 Hz, (DU20, DU24) | Non - EA | 1. l (7d)  2. m (7d)  3. k (7d) | 1. p＜0.01  2. p＜0.01  3. p＜0.01 | peri-infarct hippocampal |
| Lin 2015  (38) | SD rats  (Male, 24 / 24） | 250 ± 280 g | MCAO / R | EA 30 min after reperfusion  per day, with frequency of 5Hz / 20 Hz and  current density of 1-3 mA, (DU20, DU24). | Non - EA | 1. a (7d);  2. g (7d);  3. l (7d);  4. m (7d);  5. n (7d); | 1. p＜0.05;  2. p＜0.05;  3. p＜0.05;  4. p＜0.05;  5. p＜0.05; | ischemic brain hippocampus |
| Liu 2005  (56) | Wistar rats  (male, 14 / 15) | 340 ± 40 g | MID | MA 30 min after the completion of modeling per day, (CV17, CV12, CV6, SP10, ST36) | Sham - MA | 1. m | 1.p＜0.05 | hippocampi |
| Sun 2022  (39) | SD rats  (10 / 11) | 180 - 210 g | PSD | MA 30 min on the fourth day after successful modelling, (CV14, GV26, GV20, GV23) | Non - MA | 1. a  2. m  3. p | 1. p＜0.05;  2. p＜0.05;  3. p＜0.05; | Serum and hippocampus |
| Zheng 2020  (51) | SD rats  （Male, 10 / 10） | 270 ± 20 g | MCAO | EA 10 min after surgery  per day, with frequency of 2Hz and  current density of 1mA, (GV20, EX-HN3). | Non - EA | 1. p | 1. p＜0.05 | hippocampal slices |
| Tao 2015  (35) | SD rats  （Male, 16 / 16） | 240 - 280 g | MCAO / R | EA 30 min after reperfusion  per day, with frequency of 1Hz / 20Hz and  current density of 1mA, (LI11, ST36). | Non - EA | 1. a (3d)  2. g (3d)  3. p (3d) | 1. p <0.05;  2. p <0.05;  3. P <0.05; | lesion boundary region of the ischemic hemisphere （cortex and striatum) |
| Kim 2012  (50) | SD rats  （Male, 12 / 12） | - | MCAO / R | EA after surgery  per day, with frequency of 3Hz, (GV20, GB7) | Non - EA | 1. e (14d)  3. p (14d) | 1. p＜0.05; 2. p＜0.05; | brain tissue |
| Chen 2012  (36) | SD rats  （Male, 8 / 8） | 250 g | MCAO / R | EA 30 min after reperfusion  per day, with frequency of 1Hz / 20 Hz, (LI11, ST36) | Non - EA | 1. a (1d) 2. p (1d) | 1. p＜0.05; 2. p＜0.01; | serum |

**Note:** Model (method): MCAO (Middle Cerebral Artery Occlusion), MCAO/R (Middle Cerebral Artery Occlusion/Reperfusion), ICH (Intracerebral Hemorrhage), 4-VO (Four-Vessel Occlusion), MID (Multi-Infarct Dementia), PSD (Post-Stroke Depression). Treatment group (Method to acupuncture): EA (Electroacupuncture), MA (Manual Acupuncture), p-EA (Preconditioning Electroacupuncture), SA (Sham Acupuncture), Moxi (Moxibustion). Outcome Index (time): a. NSF (Longa) b. NSF (Longa mNSS) c. NSF (Ludmila Belayev) d. NSF (Zausinger) e. NSF (Garcia) f. NSF (Bederson) g. Infarct volume h. iron i. FTH1 j. TfR1 k. ROS l. MDA m. SOD n. GSH o.GPX4 p. BDNF q. ACSL4. NR: no report.
