# Supplementary material for: Experimental Evidence on Acupuncture Targeting Ferroptosis for Neurological Function Improvement in Cerebral Stroke: A Systematic Review and Meta‐Analysis
Source: Brain Behav. 2025 Aug 21;15(8):e70507. doi: 10.1002/brb3.70507 (PMC12370861; doi:10.1002/brb3.70507)
Supplement: Supplementary file 2 — Table s2 Subgroup analyses for the effect of acupuncture on ferroptosis after stroke. [file BRB3-15-e70507-s001.docx]

| MDA | | | | | GSH | | | | GPX4 | | | | iron | | | |
| --- | --- | --- | --- | --- | --- | --- | --- | --- | --- | --- | --- | --- | --- | --- | --- | --- |
| Groups | n | *I^2^* (*%*) | SMD [95%CI] | *P* | n | *I^2^* (*%*) | SMD [95%CI] | *P* | n | *I^2^* (*%*) | SMD [95%CI] | *P* | n | *I^2^* (*%*) | SMD [95%CI] | *P* |
| All trials | 22 | 90.5 | -3.73[-4.43, -3.03] | <0.000 | 11 | 92.7 | 3.43[2.40, 4.45] | P<0.000 | 11 | 85.4 | 2.97[2.33, 3.61] | P<0.000 | 17 | 80.1 | -3.07[-3.59, -2.55] | P<0.000 |
| Species |  |  |  | =0.989 |  |  |  | =0.229 |  |  |  | =0.012 |  |  |  | =0.002 |
| SD rats | 20 | 91.0 | -3.74[-4.48, -2.99] | <0.000 | 8 | 93.2 | 3.30[1.90, 4.16] | P<0.000 | 9 | 93.0 | 8.64[5.72, 11.56] | <0.000 | 14 | 78.9 | -2.82[-3.34, -2.31] | <0.000 |
| Wistar rats | 2 | 89.2 | -3.76[-7.31, -0.22] | =0.002 | 3 | 87.2 | 4.77[2.17, 7.36] | P<0.000 | 2 | 0.0 | 4.59[3.36, 5.82] | =0.739 | 3 | 0.0 | -4.47[-5.37, -3.57] | =0.002 |
| Weight (gram） |  |  |  | =0.069 |  |  |  | <0.000 |  |  |  | <0.000 |  |  |  | =0.002 |
| >= 300 | 1 | 0.0 | -2.56[-3.67, -1.46] | <0.000 | 0 | / | / | / | 0 | / | / | / | 3 | 0.0 | -4.47[-5.37, -3.57] | =0.515 |
| < 300 | 21 | 91.0 | -3.79[-4.53, -3.06] | <0.000 | 11 | 92.7 | 3.43[2.40, 4.45] | <0.000 | 11 | 92.7 | 3.43[2.40, 4.45] | <0.000 | 14 | 78.9 | -2.82[-3.34, -2.31] | <0.000 |
| Model |  |  |  | =0.545 |  |  |  | =0.818 |  |  |  | =0.035 |  |  |  | =0.224 |
| MCAO | 0 | / | / | / | 0 | / | / | / | 1 | 0.0 | 5[2.74, 7.26] | <0.000 | 0 | / | / | / |
| MCAO/R | 15 | 87.9 | -3.56[-4.32, -2.81] | <0.000 | 8 | 93.2 | 3.32[2.16, 4.48] | <0.000 | 2 | 0.0 | 4.59[3.36, 5.82] | =0.739 | 9 | 84.9 | -2.81[-3.44, -2.19] | <0.000 |
| ICH | 7 | 94.3 | -4.15[-5.88, -2.41] | <0.000 | 3 | 91.3 | 3.65[1.14, 6.16] | <0.000 | 8 | 93.9 | 9.28[5.95, 12.61] | <0.000 | 8 | 65.7 | -3.49[-4.38, -2.60] | =0.005 |
| Intervention time |  |  |  | =0.185 |  |  |  | =0.663 |  |  |  | =0.003 |  |  |  | 0.183 |
| 6 hours | 1 | 0 | -4.82[-6.86, -2.78] | <0.000 | 0 | / | / | / | 1 | 0.0 | 2.72[1.31, 4.12] | <0.000 | 1 | 0.0 | -1.67[-2.82, -0.51] | <0.000 |
| 1 day | 5 | 92.7 | -2.7[-3.75, -1.66] | <0.000 | 3 | 85.2 | 3.18[1.49, 4.86] | =0.001 | 3 | 95.1 | 10.61[4.09, 17.13] | <0.000 | 3 | 20.5 | -2.92[-3.47, -2.36] | =0.284 |
| 3 days | 6 | 94.5 | -4.79[-6.93, -2.65] | <0.000 | 3 | 92.6 | 2.92[0.56, 5.29] | <0.000 | 5 | 87.8 | 6.13[3.47, 8.80] | <0.000 | 5 | 28.2 | -3.09[-3.63, -2.55] | =0.233 |
| 7 days | 9 | 91.2 | -3.76[-4.89, -2.63] | <0.000 | 5 | 94.8 | 4.14[2.2.8, 6.00] | <0.000 | 2 | 88.5 | 12.53[4.33, 20.74] | =0.003 | 6 | 83.0 | -3.54[-4.59, -2.50] | <0.000 |
| 14 days | 1 | 0 | -4.00[-4.81, -3.19] | <0.000 | 0 | / | / | / | 0 | / | / | / | 2 | 93.3 | -2.75[-5.85, 0.35] | <0.000 |
| Therapeutic method |  |  |  | =0.000 |  |  |  | =0.015 |  |  |  | =0.003 |  |  |  | =0.788 |
| EA | 9 | 84.9 | -3.77[-4.86, -2.68] | <0.000 | 8 | 93.2 | 3.26[2.07, 4.45] | <0.000 | 4 | 36.1 | 4.07[3.00, 5.13] | =0.196 | 10 | 84.2 | -2.94[-3.61, -2.27] | <0.000 |
| MA | 12 | 92.9 | -3.92[-4.93, -2.90] | <0.000 | 2 | 66.6 | 4.79[2.98, 6.60] | =0.083 | 7 | 94.2 | 10.64[6.43, 14.86] | <0.000 | 6 | 59.5 | -3.31[ -4.12, -2.50] | =0.030 |
| Moxi | 1 | 0 | -1.74[-2.48, -1.01] | <0.000 | 1 | 0.0 | 2.09[1.31, 2.87] | <0.000 | 0 | / | / | / | 1 | 0.0 | -3.11[-4.04, -2.17] | <0.000 |
| Intergroup differences |  |  |  | =0.466 |  |  |  | =0.046 |  |  |  | =0.041 |  |  |  | =0.002 |
| *P*<0.01 | 11 | 83.3 | -4.00[-4.95, -3.04] | <0.000 | 4 | 94.8 | 6.26[2.52, 10.01] | <0.000 | 7 | 61.5 | 4.75[3.54, 5.96] | =0.016 | 11 | 49.5 | -3.58[-4.11, -3.05] | =0.031 |
| *P*<0.05 | 11 | 93.4 | -3.48[-4.50, -2.45] | <0.000 | 7 | 89.6 | 2.33[1.42, 3.24] | <0.000 | 4 | 96.9 | 15.22[5.25, 15.18] | <0.000 | 6 | 80.3 | -2.29[-2.91, -1.67] | <0.000 |

**Supplementary Table 2.** Subgroup analyses for the effect of acupuncture on ferroptosis after stroke. Note: *P* values for tests of subgroup differences.
