# Supplementary material for: Experimental Evidence on Acupuncture Targeting Ferroptosis for Neurological Function Improvement in Cerebral Stroke: A Systematic Review and Meta‐Analysis
Source: Brain Behav. 2025 Aug 21;15(8):e70507. doi: 10.1002/brb3.70507 (PMC12370861; doi:10.1002/brb3.70507)
Supplement: Supplementary file 3 — Table s3 Search Strategy. [file BRB3-15-e70507-s004.docx]

**Supplementary Table 3.** Search Strategy

| Search Strategy (PubMed) | |
| --- | --- |
| #1 | Acupuncture [Mesh] |
| #2 | Electroacupuncture [Title/Abstract] |
| #3 | Manual Acupuncture [Title/Abstract] |
| #4 | Scalp Acupuncture [Title/Abstract] |
| #5 | Moxibustion [Title/Abstract] |
| #6 | #1 OR #2 OR #3 OR #4 OR #5 |
| #7 | Cerebral Stroke [Mesh] |
| #8 | Strokes [Title/Abstract] |
| #9 | Cerebrovascular Accident* [Title/Abstract] |
| #10 | Cerebral Stroke* [Title/Abstract] |
| #11 | Stroke*, Cerebral [Title/Abstract] |
| #12 | Cerebrovascular Apoplexy [Title/Abstract] |
| #13 | Apoplexy, Cerebrovascular [Title/Abstract] |
| #14 | Vascular Accident, Brain [Title/Abstract] |
| #15 | Brain Vascular Accident* [Title/Abstract] |
| #16 | Vascular Accidents, Brain [Title/Abstract] |
| #17 | Cerebrovascular Stroke* [Title/Abstract] |
| #18 | Stroke* Cerebrovascular [Title/Abstract] |
| #19 | Apoplexy [Title/Abstract] |
| #20 | Stroke, Acute [Title/Abstract] |
| #21 | Acute Stroke* [Title/Abstract] |
| #22 | Cerebrovascular Accident*, Acute [Title/Abstract] |
| #23 | Acute Cerebrovascular Accident* [Title/Abstract] |
| #24 | #10 OR #11 OR #12 OR #13 OR #14 OR #15 OR #16 OR #17 OR #18 OR #19 OR #20 OR #21 OR #22 OR #23 OR #24 OR #25 OR #26 OR #27 |
| #25 | Ferroptosis [Mesh] |
| #26 | Oxytosis [Title/Abstract] |
| #27 | Iron dyshomeostasis [Title/Abstract] |
| #28 | Iron overload [Title/Abstract] |
| #29 | Iron deposition [Title/Abstract] |
| #30 | Lipid Peroxidation [Title/Abstract] |
| #31 | #25 OR #26 OR #27 OR #28 OR #29 OR #30 |
| #32 | #6 AND #24 AND #31 |
