# Supplementary material for: Experimental Evidence on Acupuncture Targeting Ferroptosis for Neurological Function Improvement in Cerebral Stroke: A Systematic Review and Meta‐Analysis
Source: Brain Behav. 2025 Aug 21;15(8):e70507. doi: 10.1002/brb3.70507 (PMC12370861; doi:10.1002/brb3.70507)
Supplement: Supplementary file 4 — Table s4 Trim‐And‐Fill Analysis Results. [file BRB3-15-e70507-s002.docx]

| **index** | ***t*-values** | ***p*-values** | **trimmed studies added** | **original SMD** | **adjusted SMD** |
| --- | --- | --- | --- | --- | --- |
| MDA^**^ | -5.51 | 0.000 | 4 | -3.73 | -3.12 |
| GSH^**^ | 7.24 | 0.000 | 3 | 3.43 | 2.84 |
| GPX4^**^ | 12.58 | 0.000 | 2 | 2.97 | 2.52 |
| iron^**^ | -4.01 | 0.001 | 1 | -3.07 | -2.65 |

**Supplementary Table 4.** Trim-And-Fill Analysis Results.
